# New bitongling regulates gut microbiota to predict angiogenesis in rheumatoid arthritis via the gut-joint axis: a deep neural network approach

**DOI:** 10.3389/fmicb.2025.1528865

**Published:** 2025-02-03

**Authors:** Yin Guan, Xiaoqian Zhao, Yun Lu, Yue Zhang, Yan Lu, Yue Wang

**Affiliations:** ^1^Department of Rheumatism Immunity Branch, Affiliated Hospital of Nanjing University of Chinese Medicine, Nanjing, China; ^2^Department of Ethics Committee, Affiliated Hospital of Nanjing University of Chinese Medicine, Nanjing, China

**Keywords:** rheumatoid arthritis (RA), new bitongling (NBTL), gut microbiota, deep neural network, angiogenesis

## Abstract

**Background:**

Rheumatoid arthritis (RA) is a persistent autoimmune disorder marked by inflammation and joint damage. Although current treatments, such as disease-modifying antirheumatic drugs (DMARDs), help control symptoms, they frequently cause substantial side effects, highlighting the urgent need for safer and more effective alternatives. Recent research indicates that gut microbiota might be pivotal in RA development through the “gut-joint axis,” presenting novel therapeutic possibilities.

**Purpose:**

This study seeks to explore the therapeutic potential of the traditional Chinese medicine (TCM) compound new bitongling (NBTL) for RA, with an emphasis on its capacity to regulate gut microbiota and suppress angiogenesis via the vascular endothelial growth factor (VEGF) signaling pathway.

**Methods:**

We utilized a collagen-induced arthritis (CIA) rat model to assess the impact of NBTL. The study employed 16S ribosomal DNA (16S rDNA) sequencing to analyze gut microbiota composition, machine learning techniques to identify characteristic microbial taxa, and transcriptomic analysis (GSVA) to assess the impact on the VEGF signaling pathway. The findings were further validated through analysis with deep neural network models and *in vivo*/*in vitro* experiments, including western blot, immunofluorescence, and miRNA analysis.

**Results:**

NBTL treatment markedly diminished inflammation in RA rats, evidenced by the reduced expression of TNF-α, IL-17, IL-6, and ASC in synovial tissues. Histopathological analysis confirmed alleviation of joint damage. Five characteristic microbial taxa, including *f_Mycoplasmataceae*, *s_Metamycoplasma_sualvi*, and *g_Prevotellaceae_Ga6A1_group*, were identified and associated with NBTL’s modulation of the VEGF pathway. Gene set variation analysis (GSVA) revealed significant downregulation of the VEGF signaling pathway following NBTL treatment. Subsequent experiments confirmed that NBTL inhibited VEGF and its receptors, VEGFR1 and VEGFR2, along with HIF-1α (hypoxia-inducible factor 1-alpha), thereby reducing angiogenesis. Additionally, NBTL upregulated miR-20a-5p and miR-223-3p, contributing to its anti-angiogenic effects.

**Conclusion:**

NBTL exhibits significant therapeutic potential in RA by modulating gut microbiota and inhibiting the VEGF signaling pathway. These findings support NBTL’s use as a promising candidate for RA treatment, emphasizing the need for further research on its mechanisms and clinical application.

## 1 Introduction

Rheumatoid arthritis (RA) is an autoimmune disorder mainly defined by inflammation in the synovial joints, resulting in swelling, pain, and permanent joint damage. This significantly impairs patients’ quality of life and work capacity. Research indicates that within 10 years, the disability rate for RA patients can reach as high as 43.5%. As the number of cases continues to rise, RA has become a major rheumatic disease globally, with a prevalence of 0.5 to 1% (Almutairi et al., 2021). Although RA remains incurable, guidelines from major rheumatology associations in western countries recommend a treat-to-target strategy to alleviate symptoms and control disease progression (Aletaha et al., 2010). However, prolonged use of existing treatments, including conventional and biologic disease-modifying antirheumatic drugs (DMARDs), is frequently associated with significant side effects (El Masri et al., 2020; Gomides et al., 2021). Consequently, there is a pressing need to explore new, effective treatments with fewer adverse effects.

Recent research has increasingly explored the possible connection between gut microbiota and RA. Epidemiological and translational studies have indicated that imbalances in gut microbiota are strongly linked to RA development (Catrina et al., 2016; Scher et al., 2016; Scher et al., 2013; Holers et al., 2018). Notably, gut microbiota composition shifts significantly in the early stages of RA, contributing to the “gut-joint axis” theory (Wells et al., 2020; Zaiss et al., 2021). This theory posits that dysbiosis in gut microbiota not only promotes the onset of RA but also contributes significantly to maintaining the inflammatory state associated with the disease (Zhang X. et al., 2015; Chen et al., 2016; Marietta et al., 2016; Maeda et al., 2016; Alpizar-Rodriguez et al., 2019; Inamo, 2021; Alpizar Rodriguez et al., 2021; Jeong et al., 2019). Additionally, angiogenesis in the synovium, which provides nutrients to inflamed areas and exacerbates joint damage, is also linked to microbial imbalances (Zaiss et al., 2021; Zhang Y. et al., 2015; Alunno et al., 2015; Rogier et al., 2017). These findings offer new perspectives on RA pathophysiology and suggest the potential for therapeutic strategies targeting gut microbiota.

One research has shown that pairing mangiferin with cinnamic acid can ease RA symptoms through inhibition of the TLR4/NFκB/NLRP3 signaling pathway (Li et al., 2022). Various bioactive compounds derived from TCM, such as alkaloids, saponins, flavonoids, and terpenoids, have also shown potential in suppressing bone destruction and modulating immune responses (Shi et al., 2020). Furthermore, TCM components have been found to reduce RA symptoms by regulating endoplasmic reticulum stress, providing a basis for new therapeutic approaches (de Seabra Rodrigues Dias et al., 2021). Meta-analyses and clinical evaluations have further emphasized the effectiveness of TCM formulas in improving key biomarkers, such as rheumatoid factor and anti-cyclic citrullinated peptide antibodies, in RA patients (Tang et al., 2021). These results offer solid support for incorporating TCM into modern medical practices.

Importantly, recent studies have emphasized TCM’s considerable potential in modulating gut microbiota for RA treatment. For example, studies have shown that Danggui Niantong Decoction can improve RA by regulating gut microbiota and promoting mitochondrial apoptosis in animal models (Lu et al., 2022). Similarly, paeoniflorin has been shown to dynamically reshape gut microbiota in CIA rats, indicating its potential therapeutic effects on RA through microbiota modulation (Peng et al., 2019). These studies underscore the importance of exploring TCM’s role in modulating gut microbiota as a therapeutic strategy for RA.

In addition to gut microbiota regulation, TCM has demonstrated significant potential in inhibiting angiogenesis related to RA. For example, Yuxuebi tablets have demonstrated anti-angiogenic activity in RA rat models by inhibiting the LOX/Ras/Raf-1 signaling pathway (Su et al., 2022). Similarly, oxymatrine has been found to inhibit synovial angiogenesis in CIA rats by targeting the HIF-VEGF-Ang signaling and the PI3K/Akt pathways (Ao et al., 2022). These pieces of evidence further reinforce the potential of targeting angiogenesis to control RA progression.

Based on this background, our study hypothesizes that new bitongling (NBTL) can modulate gut microbiota to influence the VEGF pathway, thereby inhibiting angiogenesis in RA and achieving therapeutic effects. Our research team has previously demonstrated that new bitongling significantly improves the pathological state of RA in rats and alleviates arthritis symptoms by inhibiting the JAK2/STAT3 signaling pathway (Li et al., 2021). To gain deeper insights into the mechanism, we intend to apply deep neural network prediction techniques to construct a regulatory model linking gut microbiota and the VEGF pathway. Experimental validation of the model’s predictions will provide robust evidence for NBTL’s efficacy in treating RA, furthering the integration of TCM into modern medicine.

## 2 Materials and methods

### 2.1 Preparation of new bitongling and Tripterygium wilfordii tablets

New bitongling, a traditional Chinese medicine (TCM) compound, is formulated using herbs such as *Cinnamomi ramulus* (*Gui Zhi*), *Saposhnikoviae radix* (*Fang Feng*), *Ephedrae herba* (*Ma Huang*), *Sinomenii caulis* (*Qing Feng Teng*), *Aconiti radix* (*Chuan Wu*), and *Vespae nidus* (*Feng Fang*), sourced from the pharmacy at Jiangsu Provincial Hospital of TCM. Prior to preparation, all raw herbs underwent stringent quality control measures, including morphological identification and visual inspection to verify the authenticity and purity of each herb. Additionally, each herb was standardized based on established pharmacopeial standards (China Pharmaceutical Association, 2020), ensuring that they met predefined criteria for key active components to guarantee consistency across different batches.

The preparation process involves several key steps: *Aconiti radix* (Chuan Wu) is first decocted in double-distilled water at 100°C for 30 min. Subsequently, the remaining herbs are added and the mixture is decocted for an additional 45–60 min. The resulting liquid is then filtered to obtain the herbal extract. This decoction process is repeated to ensure thorough extraction of active constituents. The combined extracts are concentrated and centrifuged at 5,000 rpm for 30 min to eliminate residues, yielding a final concentration of 1.22 g/mL. The decoction is then aliquoted and stored at −20°C for future use. All preparation batches underwent quality control checks, including visual inspection and adherence to standardized preparation protocols, to confirm the consistency and potency of the extracts.

For the positive control, Tripterygium wilfordii tablets containing 10 mg of the active ingredient were used. These tablets, produced by Zhejiang Deyongde Pharmaceutical Co., Ltd. (Batch number: 2204111B), were ground into a fine powder and dissolved in distilled water to create a suspension. The suspension was standardized to a final concentration of 8 mg/mL through precise measurement and preparation techniques to ensure the stability and uniformity of the active components for experimental use.

### 2.2 Selection of experimental animals, housing, model construction, and grouping

In this study, we selected 6-week-old female Sprague–Dawley (SD) rats, weighing between 180–200 g, sourced from Zhejiang Vital River Laboratory Animal Technology Co., Ltd. (License No. SCXK(Zhe)2021-0006). The rats were acclimatized for 1 week before the experiment in a specific pathogen-free (SPF) facility at the Affiliated Hospital of Nanjing University of Chinese Medicine (License No. SYXK(Su)2017-0069), maintained under controlled conditions (22 ± 2°C, 45–60% humidity). They had free access to SPF-grade feed (Synergy Bio, 1010086) and water. The study adhered to National Institutes of Health (NIH) guidelines for the care and use of laboratory animals and received approval from the Ethics Committee of the Affiliated Hospital of Nanjing University of Chinese Medicine (Approval No. 2022 DW-37-01).

For arthritis induction, bovine type II collagen (CII) (Beijing Bolide Biotech, 20021) was dissolved in 0.1 mol/L glacial acetic acid to a concentration of 4 g/L, stirred on ice, and stored overnight at 4°C. This solution was then emulsified with an equal volume of complete Freund’s adjuvant (CFA) (Sigma, F5881) to create a 2 g/L collagen II emulsion. The rats were randomly assigned to either a normal group or a collagen-induced arthritis (CIA) model group. The CIA induction protocol involved intradermal injections of 0.3 mL of the CII emulsion on day 1, followed by a booster dose on day 7. The primary immunization consisted of a 1:1 mix of bovine type II collagen and CFA, administered at a 2 g/L concentration. The booster immunization used a similar 1:1 mix with incomplete Freund’s adjuvant (IFA) (Sigma, F5506). Arthritis severity was evaluated on day 14 using a visual scoring system for each paw (0–4, with a maximum total score of 16): 0 for normal joints, 1 for slight swelling or erythema, 2 for moderate erythema and swelling, 3 for pronounced erythema and swelling, and 4 for severe swelling, erythema, or ankylosis (Trentham et al., 1977).

The rats with successfully induced arthritis were randomly allocated into different treatment groups, along with a blank control group selected from the normal group. The groups received the following treatments via oral gavage for 28 days (Li et al., 2021): blank control group received distilled water; model group received distilled water; low dose new bitongling group received 2.75 g/kg/day of new bitongling; medium dose new bitongling group received 5.5 g/kg/day of new bitongling; high dose new bitongling group received 11 g/kg/day of new bitongling; and the positive control group received 0.008 g/kg/day of Tripterygium wilfordii solution. The selected doses for the low, medium, and high new bitongling (NBTL) groups were based on dose extrapolation from equivalent human doses using the standard body surface area (BSA) conversion formula (Nair and Jacob, 2016; Reagan-Shaw et al., 2008). These doses were further refined through preliminary experiments reported in our prior study, which demonstrated the efficacy and safety of these specific concentrations in a similar model (Li et al., 2021). The rats were monitored daily for changes in appearance, behavior, food intake, and excretion, with body weight measurements taken weekly. After 28 days, the rats were anesthetized for blood serum collection and then euthanized for the collection of ankle and knee synovial tissue.

### 2.3 Preparation of serum containing new bitongling and control serum

We selected 6–8 week-old SPF-grade SD rats and randomly divided them into the traditional Chinese medicine (TCM) group and the blank control group. The TCM group received an oral gavage of new bitongling (NBTL) at the optimal dose for *in vivo* rat experiments (high dose: 11 g/kg/day, with a drug concentration of 1.22 g/mL). The blank control group was administered an equal volume of physiological saline. Both treatments were administered daily for seven consecutive days. One hour after the final administration, the rats were anesthetized, and blood samples were collected. The blood was allowed to clot at 4°C for 2 h to facilitate serum and clot separation. The samples were then centrifuged at 3,000 rpm for 10 min to separate the serum from the clot. The supernatant, which is the serum, was carefully collected. The collected serum was then inactivated at 56°C to eliminate any potential biological activity. This step is performed after serum separation. Following inactivation, the serum was filtered for sterilization, aliquoted, and stored at −80°C for subsequent experiments (Pan et al., 2023; Pei et al., 2024).

### 2.4 Identification of prototypical components and metabolites of new bitongling in blood using UPHLC-MS-TOF

We used ultra-performance high-performance liquid chromatography coupled with time-of-flight mass spectrometry (UPHLC-MS-TOF) to identify the prototypical components and metabolites of new bitongling (NBTL) in rat serum. Serum samples were prepared by protein precipitation and filtration. Chromatographic separation was performed on an acquity UPLC BEH C18 column, followed by mass spectrometric analysis with an electrospray ionization source in both positive and negative ion modes. Data processing included peak detection, alignment, and compound identification through database matching.

Ion peak data were filtered based on various conditions, with missing values removed. Positive and negative ion data were processed separately and converted to a long format for plotting, retaining only NBTL medicated serum group data. Vertical offsets were applied to avoid overlapping annotations. The “ggplot2” package (Villanueva and Chen, 2019) was used to visualize the data, creating plots for positive and negative ions to illustrate the prototypical components and metabolites.

### 2.5 Hematoxylin and eosin staining of synovial tissue

Synovial tissue samples were fixed in 4% paraformaldehyde (PFA) (Aladdin, C104188; Sinopharm, 30525-89-4), dehydrated through graded ethanol solutions (Sinopharm, 100092683), and embedded in paraffin. Sections were cut at 4 microns, dewaxed, and rehydrated. Hematoxylin staining (Zhuhai Beisuo, BA4097) was used to highlight cell nuclei, followed by eosin staining (Zhuhai Beisuo, BA4099) to highlight cytoplasm. The sections were then dehydrated with anhydrous ethanol (Sinopharm, 100092683), cleared with xylene (Sinopharm, 1330-20-7), and mounted with neutral balsam (Sinopharm, 10004160). Hematoxylin and eosin (HE) staining was evaluated for synovial cell arrangement, hyperplasia, and inflammatory cell infiltration, with scores ranging from 0 (no lesion) to 4 (very severe) based on the extent of disorganization, hyperplasia, and infiltration. The average score was calculated to assess tissue damage severity across samples (Guo et al., 2017).

### 2.6 Serum enzyme-linked immunosorbent assay analysis

Serum samples stored at −80°C were thawed to room temperature for enzyme-linked immunosorbent assay (ELISA) analysis. The following reagents and consumables were used: rat interleukin-6 (IL-6) ELISA kit (CSB-E04640r), rat interleukin-17 (IL-17) ELISA kit (CSB-E07451r), and rat tumor necrosis factor alpha (TNF-α) ELISA kit (CSB-E11987r) from Cusabio. Biotin-labeled antibodies and HRP-labeled streptavidin were diluted with their respective diluents at a ratio of 10 μL to 990 μL and kept on ice. The wash buffer was prepared by mixing 1 mL of concentrate with 24 mL of deionized water. Standard proteins were prepared through serial dilutions. The ELISA procedure involved adding standard proteins and serum samples to a coated 96-well plate, followed by incubation at 37°C. After 2 h, 100 μL of biotin-labeled antibody was added and incubated for another hour. The plate was then washed five times with 200 μL of wash buffer per wash, followed by the addition of 100 μL of HRP-labeled streptavidin and a 1-h incubation. The wash step was repeated, and 90 μL of 3,3′,5,5′-tetramethylbenzidine (TMB) substrate was added and incubated in the dark. Finally, 50 μL of stop solution was added, and the absorbance was measured at 450 nm using a microplate reader.

### 2.7 Western blot analysis

In this study, western blot analysis was performed on synovial tissue samples. Key reagents included 10X Transfer Buffer, 10X Electrophoresis Buffer (pH 8.3), and 10X Tris Buffered Saline with Tween-20 (TBST) Buffer. We prepared working solutions such as 10% sodium dodecyl sulfate (SDS), 10% ammonium persulfate (APS), and Tris-HCl buffers at pH 8.8 and 6.8, along with stacking and separating gels of varying concentrations for sodium dodecyl sulfate-polyacrylamide gel electrophoresis (SDS-PAGE). The synovial tissues were minced, ground in liquid nitrogen, and lysed using radioimmunoprecipitation assay (RIPA) buffer with a protease inhibitor cocktail (Sigma, P8340). Protein concentrations were determined using the bicinchoninic acid (BCA) protein assay kit (Beyotime, P0010). The samples were resolved on SDS-PAGE gels and transferred to polyvinylidene difluoride (PVDF) membranes (Millipore, ISEQ00010). Membranes were blocked with 5% skim milk (BD, 232100) in TBST, then incubated overnight with primary antibodies in 1% bovine serum albumin (BSA)/TBST (Sigma-Aldrich, V900933). The primary antibodies used were glyceraldehyde 3-phosphate dehydrogenase (GAPDH) (Zen-Bio, 200306-7E4), vascular endothelial growth factor (VEGF) (Santa Cruz, sc-53463), vascular endothelial growth factor receptor 1 (VEGFR1) (Proteintech, 19003-1-AP), vascular endothelial growth factor receptor 2 (VEGFR2) (Abcam, ab32152), hypoxia-inducible factor 1-alpha (HIF-1α) (Santa Cruz, SC-13515), and apoptosis-associated speck-like protein containing a CARD (ASC) (Santa Cruz, sc-514414). Secondary antibodies included HRP-conjugated anti-mouse (Beyotime, A0216) and anti-rabbit (Beyotime, A0208), as well as fluorescent antibodies Alexa Fluor 488 Donkey Anti-Rabbit IgG (H + L) (Abcam, ab150061) and Alexa Fluor 488 Donkey Anti-Mouse IgG (H + L) (Abcam, ab150105). Detection was performed using an enhanced chemiluminescence (ECL) substrate (Tanon, 180-5001) and visualized with a Tanon5200 Imaging System.

### 2.8 Quantitative polymerase chain reaction for mRNA and miRNA analysis in rat and cell samples

For quantitative polymerase chain reaction (qPCR) detection of mRNA and miRNA in rats and cells, we use specific kits and reagents. RNA is extracted using RNAiso Plus from Takara, combined with Alladin’s chloroform, isopropanol, ethanol, and diethylpyrocarbonate (DEPC) (C128130, I112011, E111989, D105557). This involves homogenizing in TRIzol (Thermo Fisher Scientific, 15596026), adding chloroform, and undergoing centrifugation to isolate RNA, which is then precipitated and washed before dissolving in DEPC water for concentration measurement.

For reverse transcription, the PrimeScript^™^ RT kit with gDNA Eraser from Takara is used for mRNA, while miRNA in rats employs the Ribobio’s Bugle-Loop^™^ RT kit (Guangzhou RiboBio Co., Ltd.) (Zhou et al., 2015), following the specific protocols of each. Quantitative PCR is performed using SYBR^®^ Premix Ex Taq^™^ from Takara, starting with a 95°C denaturation, followed by 40 cycles and melting curve analysis.

Expression levels are calculated using the $2^{-\mathrm{\Delta \Delta CT}}$ method, comparing Ct values between target and reference genes (Livak and Schmittgen, 2001). Rat mRNA primers are detailed in Table 1. Due to intellectual property constraints, rat miRNA primer sequences from the Ribobio kit are not disclosed. Cell miRNA primers are provided in Table 2.

**Table 1:** mRNA gene primer sequences.

Primer name	Primer sequence (5′–3′)
rno-VEGF-F	ACAGAAGGGGAGCAGAAAGC
rno-VEGF-R	GCTGGCTTTGGTGAGGTTTG
rno-ASC-F	GCCATGGACCTCACTGACAA
rno-ASC-R	TTGGTGGTCTCTGCTCGAAC
rno-GAPDH-F	TTCACCACCATGGAGAAGGC
rno-GAPDH-R	AGTGATGGCATGGACTGTGG

**Table 2:** miRNA gene primer sequences.

Primer name	Primer sequence (5′–3′)
hsa-miR-223-3p-RT	CTCAACTGGTGTCGTGGAGTCGGCAATTCAGTTGAGTGGGGTAT
hsa-miR-223-3p-F	ACACTCCAGCTGGGTGTCAGTTTGTCAAAT
hsa-miR-20a-5p-RT	CTCAACTGGTGTCGTGGAGTCGGCAATTCAGTTGAGCTACCTGC
hsa-miR-20a-5p-F	ACACTCCAGCTGGGTAAAGTGCTTATAGTGC
universal primer -A	TGGTGTCGTGGAGTCG
U6-F	CTCGCTTCGGCAGCACA
U6-R	AACGCTTCACGAATTTGCGT

### 2.9 Gut microbiota 16S rDNA sequencing of rats

Rats were anesthetized with 10% Sodium Pentobarbital (Wengjiang Reagent, CAS: 57-33-0) via intraperitoneal injection. Fecal samples were collected under sterile conditions and either used immediately or stored at −80°C. Microbial DNA was extracted using the E.Z.N.A.^®^ Soil DNA Kit (Omega Bio-tek, United States), with quality checked by agarose gel electrophoresis. Concentration and purity were measured with a NanoDrop2000 spectrophotometer (Thermo Scientific, United States). The V3–V4 region of the 16S rRNA gene was amplified using primers 338F and 806R. The PCR mix included TransStart FastPfu Buffer, dNTPs, primers, and DNA polymerase, in a final volume of 20 μL. The PCR process began with denaturation at 95°C for 3 min, followed by 27 cycles at 95°C, 55°C, and 72°C, each for 30 s. The final step was a 10-min extension at 72°C, followed by cooling at 4°C. PCR products were separated on a 2% agarose gel, then purified and quantified. Sequencing libraries were created using the NEXTFLEX Rapid DNA-Seq Kit, which included adapter ligation, magnetic bead purification, and PCR amplification. Sequencing was performed using the Illumina PE300/PE250 platform. The raw paired-end data underwent quality control with Fastp (Chen et al., 2018) (v0.19.6) and were merged using FLASH (Magoč and Salzberg, 2011) (v1.2.11). Clustering into operational taxonomic units (OTUs) was done with UPARSE (Edgar, 2013; Stackebrandt and Goebel, 1994) (v7.1) at 97% similarity, with chimeric and mitochondrial sequences removed. To balance sequencing depth, each sample was normalized to 20,000 reads, achieving a Good’s coverage of 99.09%. OTUs were classified with the RDP Classifier (Wang et al., 2007) (v2.11) at a 70% confidence threshold, referencing the Silva 16S rRNA Gene Database (v138), followed by community composition analysis.

### 2.10 Preliminary analysis of gut microbiota

The “data table” package (Barrett et al., 2021) was used to read the OTU table, set OTU ID as row names, and extract sample and taxonomy tables. The “amplicon” package (Callahan et al., 2016) calculated microbial relative abundance with a threshold of 1 × 10^−4^.

The “ropls” package (Thévenot et al., 2015) constructed the partial least squares discriminant analysis (PLS-DA) model, and the “ggplot2” and “ggsci” (Xiao, 2023) packages visualized the results, including sample score and variable importance plots.

To observe gut microbial abundance changes, the total abundance of each species was calculated and sorted. The top 10 species by abundance and the total abundance of remaining species were displayed using stacked bar plots with “ggplot2” package. The “aplot” package (Yu, 2023) added group information.

For LEfSe (linear discriminant analysis effect size) analysis, the OTU table was loaded with identifiers as row names. A sample table and formatted taxonomy table were created. The “microeco” package (Liu C. et al., 2021) integrated these tables into a Microtable object. LEfSe analysis was conducted using the trans_diff$new function, with bar plots and a cladogram generated for taxonomic differences. Correlation analysis with the “corrplot” package (Wei et al., 2021) produced a correlation heatmap among gut microbiota.

### 2.11 Identification and analysis of characteristic gut microbiota associated with NBTL treatment in rheumatoid arthritis using SVM-RFE and random forest

We utilized the “tidyverse” (Wickham et al., 2019) packages for data preprocessing. The support vector machine-recursive feature elimination (SVM-RFE) algorithm, implemented via the “e1071” package (Meyer et al., 2023), was employed for feature selection, identifying influential features for classification. K-fold cross-validation evaluated the feature set effectiveness, with error rates and model accuracy visualized to assess the impact of feature subsets. The best-performing subset was selected based on error rates. A random forest model, created with the “randomForest” package (Liaw and Wiener, 2002), evaluated feature importance, and its generalizability was tested with five repetitions of 10-fold cross-validation. The optimal feature subset was selected based on importance scores and error rates.

Next, key microbiota identified from the SVM-RFE and random forest analyses were extracted and compared using the “VennDiagram” package (Chen and Boutros, 2011), with overlapping microbiota exported for receiver operating characteristic (ROC) analysis. The “pROC” package (Robin et al., 2011) was used for ROC analysis, calculating the area under the curve (AUC) values. An AUC >0.7 was considered indicative of strong discriminative power for key microbiota. ROC curves were plotted and saved, and data for high AUC microbiota were exported.

Finally, the “data table” and “tidyverse” packages processed expression data of target microbiota. Boxplots were generated with the “ggsignif (Ahlmann-Eltze and Preisenergie, 2021), “ggplot2,” and “ggpubr” (Kassambara, 2023) packages to illustrate abundance differences among groups. The stat_compare_means function added *p*-values, identifying significant microbiota associated with NBTL treatment for rheumatoid arthritis.

### 2.12 Transcriptomic sequencing of rat synovial tissue mRNA

RNA was extracted from tissue samples using TRIzol^®^ Reagent (Invitrogen) and RNAiso Plus (Takara, 9108). Concentration, purity, and integrity were assessed with a NanoDrop 2000 spectrophotometer and agarose gel electrophoresis. Quality was further verified using an Agilent 2100 Bioanalyzer, ensuring ≥1 μg of total RNA, a concentration of at least 50 ng/μL, and an OD 260/280 ratio within 1.8 to 2.2. diethylpyrocarbonate (DEPC) water (Alladin, D105557) was used in all preparations to ensure RNase-free conditions. mRNA was extracted using Oligo(dT) magnetic beads, then fragmented into ~300 bp pieces with a buffer for Illumina Sequencing. Using the PrimeScript^™^ RT reagent Kit with gDNA Eraser (Takara, RR047A), double-stranded cDNA was synthesized from fragmented mRNA with random primers. The cDNA then underwent end repair, A-tailing, and Y-shaped adapter ligation using the Truseq^™^ RNA Sample Prep Kit (Illumina). Adapter-ligated cDNA was purified, size-selected, and amplified by PCR to generate the final sequencing library. The libraries were quantified with the QuantiFluor^®^ dsDNA System (Promega), and sequencing was conducted on the Illumina HiSeq X Ten platform using its reagent kit. Sequencing reads were analyzed for base distribution and quality, followed by quality control, reference genome alignment, and expression level analysis, with results reported in transcripts per million (TPM).

### 2.13 Preliminary bioinformatics analysis of the genetic basis of angiogenesis

We used the “ropls” package to create a PLS-DA model, extracting results like the model summary and VIP (variable importance in projection) values. Sample distributions and confidence ellipses were then plotted using the “ggplot2” package. Kyoto Encyclopedia of Genes and Genomes (KEGG) gene sets were acquired using the “msigdbr” package (Dolgalev, 2022) to prepare gene lists for gene set variation analysis (GSVA). The analysis was carried out with the “GSVA” package (Hänzelmann et al., 2013), followed by differential analysis using GraphPad Prism.

For weighted gene co-expression network analysis (WGCNA), we processed and cleaned the gene expression data. The “WGCNA” package (Langfelder and Horvath, 2008, 2012) was then used for clustering analysis and the removal of outlier samples. The optimal soft threshold was determined, and a co-expression network was constructed to identify modules. These modules were analyzed for their correlation with phenotypic traits, and relevant plots were generated.

Next, module genes were analyzed for protein–protein interactions (PPI) using the STRING database (Szklarczyk et al., 2019).^1^ Cytoscape (Shannon et al., 2003) was used to visualize the interaction data, and the cytoHubba plugin (Bader and Hogue, 2003) identified hub genes through degree analysis. Following this, we read and cleaned the gene expression data, imported the top 10 genes based on degree ranking, and standardized the GSVA results for the vascular endothelial growth factor (VEGF) pathway. The “vegan” package (Oksanen et al., 2022) was used for correlation analysis, and the results were visualized with the “ggcor” package (Huang et al., 2020).

We imported and cleaned the synovial PCR results and microbiota data. Spearman correlation tests were conducted to examine relationships between these datasets, and scatter plots were generated. Lastly, we imported synovial PCR data, selected samples, conducted correlation tests, and plotted scatter diagrams to display the relationship between miRNA and VEGF expression.

### 2.14 Deep neural network model construction and evaluation

Load the “data table,” “mlr3” (Lang et al., 2019), “mlr3learners” (Lang et al., 2023), “mlr3pipelines” (Binder et al., 2021), and “keras” (Chollet and Allaire, 2023) packages, and use the “reticulate” package (Kalinowski et al., 2024) to access the Keras library (Chollet, 2015) from a Python environment. Specify the working directory, load the dataset, and split it into 80% for training and 20% for testing. Standardize the training data and apply principal component analysis (PCA) for dimensionality reduction. Construct a sequential deep neural network (DNN) model with two hidden layers, using rectified linear unit (ReLU) activation and the Adam optimizer. The model was compiled using mean squared error (MSE) as the loss function and mean absolute error (MAE) as the performance metric. It was trained with a 20% validation split and evaluated on the test set. Plot comparisons of predicted versus actual values, calculate the *R*^2^ value, monitor the loss curve during training, and display a graphical representation of the model structure.

### 2.15 Cell culture and screening for the effective concentration of NBTL-containing serum

Human umbilical vein endothelial cells (HUVECs) were sourced from the American Type Culture Collection (ATCC) and stored in liquid nitrogen. Before culture, cells were quickly thawed in a 37°C water bath and transferred to a 15 mL centrifuge tube containing 10 mL of pre-warmed Roswell Park Memorial Institute (RPMI)-1640 medium (Gibco, C11875500BT) supplemented with 10% fetal bovine serum (FBS) (Gibco, 10099-141C) and 2 mM L-glutamine. After centrifugation at 1,000 rpm for 5 min, the supernatant was discarded, and the cell pellet was resuspended in fresh pre-warmed medium and transferred to a pre-coated T25 culture flask. HUVECs were maintained at 37°C in a 5% CO_2_ atmosphere, with medium changes every 2–3 days. Cells were passaged at 80–90% confluence using 0.25% trypsin-EDTA (Gibco, Wisent 325-043-EL) for 1–2 min at 37°C, followed by centrifugation at 1,000 rpm for 5 min. Digestion was halted with an equal volume of RPMI-1640 containing 10% FBS, and cells were resuspended in fresh complete medium and redistributed into new culture flasks. For cryopreservation, cells were harvested, resuspended in CELLSAVING serum-free cryopreservation solution (C40100), aliquoted into cryovials, and stored at −80°C.

To screen for the effective concentration of NBTL-containing serum, HUVECs were cultured to an appropriate density and seeded into 96-well plates at 5 × 10^3^ cells per well in RPMI-1640 medium with 10% FBS. Cells were treated with various concentrations of NBTL-containing serum (0, 2, 4, 8, 12, 16, 20%) or an equal volume of blank rat serum for 24 h at 37°C and 5% CO_2_. It is important to note that the intervention was carried out solely with rat serum and RPMI-1640 basal medium, without the addition of FBS. Each concentration was tested in triplicate, followed by the addition of 10 μL of Cell Counting Kit-8 (CCK-8) reagent (APExBIO, K1018) and 90 μL of RPMI-1640 medium, with further incubation for 1–4 h. Absorbance was measured at 450 nm using a microplate reader.

### 2.16 Scratch assay

HUVECs (1 × 10^5^ cells per well) were cultured in 6-well plates until they reached confluence. A scratch was then created using a sterile 200 μL pipette tip, followed by treatment with either NBTL-containing serum at its optimal concentration or blank rat serum. Cell migration and scratch closure were evaluated by capturing images at the beginning (0 h) and after 24 h using an inverted microscope.

### 2.17 Carboxyfluorescein diacetate succinimidyl ester flow cytometry

HUVECs (1 × 10^5^ cells per well) were seeded in 6-well plates and labeled with 10 μM carboxyfluorescein diacetate succinimidyl ester (CFSE) (Beyotime, C1031). After 15 min of incubation at 37°C in the dark, the cells were rinsed with phosphate-buffered saline (PBS) (Nanjing Shengxing, SN331). The cells were then exposed to either control rat serum or NBTL-containing serum for 24 h, collected, and resuspended in PBS. Flow cytometry was used to assess cell proliferation, utilizing a 488 nm excitation and 518 nm emission.

### 2.18 Angiogenesis assay

HUVECs (1 × 10^5^ cells per well) were first cultured in 6-well plates and treated with either control rat serum or NBTL-containing serum for 24 h. The cells were then transferred to Matrigel-coated 24-well plates (1.5 × 10^5^ cells per well) and incubated for 4 h. Tube formation was documented and quantified using ImageJ software with the Angiogenesis Analyzer plugin.

### 2.19 Immunofluorescence assay

Cells were seeded at 1 × 10^5^ density into 3.5 cm glass-bottom culture dishes and incubated overnight. The next day, the media was replaced with control or drug-containing serum based on CCK8 results, followed by a 24-h incubation. Cells were fixed with 4% paraformaldehyde (PFA) for 10 min, then washed with pre-cooled PBS and permeabilized using 0.5% Triton X-100 on ice. Blocking was performed with 3% bovine serum albumin (BSA) for 30 min at room temperature. Cells were incubated overnight at 4°C with primary antibodies (VEGF, VEGFR2, HIF-1α, VEGFR1) diluted in PBS with 1% BSA. After washing, secondary antibodies (1:400 dilution) were applied for 1 h at room temperature in the dark. Nuclei were stained with 100 ng/mL DAPI for 10 min, followed by washes. Finally, an anti-fade reagent was added, and the cells were imaged using a fluorescence microscope.

### 2.20 Statistical analysis and software utilization

In this study, the analysis and processing of gut microbiota data were primarily conducted using R software (R Core Team, 2021) (version 4.3.0). For the correlation analysis between synovial PCR results and characteristic gut microbiota, R software (version 4.3.1) was employed. The construction and training of the deep neural network (DNN) model utilized R software (version 4.3.0), while other R analyses were mainly performed using version 4.3.2. Grayscale value analysis of western blot results, scratch assay migration area analysis, and angiogenesis assay analysis were all conducted using ImageJ software. CFSE flow cytometry analysis was carried out with FlowJo software (version 10.8.1). Further data processing and graphical plotting were completed using GraphPad Prism software (version 9.5.0).

For statistical comparisons, one-way analysis of variance (ANOVA) was used for comparisons among multiple groups with a single factor, and two-way ANOVA was employed for comparisons involving two factors. Paired *t*-tests were used for within-group comparisons, while unpaired *t*-tests were employed for comparisons between independent groups. Before performing statistical tests, normality was assessed using the Shapiro–Wilk test, and homogeneity of variance was evaluated using Levene’s test. If these assumptions were met, parametric tests (ANOVA and *t*-tests) were applied. For non-normally distributed data, non-parametric tests (e.g., Mann–Whitney *U* test) were used.

In all statistical analyses, significance levels were set as follows: *p*-values under 0.05 were considered statistically significant, those below 0.01 were highly significant, and *p*-values less than 0.001 or 0.0001 were deemed extremely significant.

### 2.21 Statistical power and sample size

#### 2.21.1 Animal experiments

The experiment involved six groups: control, model, low-dose NBTL, medium-dose NBTL, high-dose NBTL, and positive control, with each group containing eight animals (*n* = 8). As stated in Trentham et al. (1977), Charan and Kantharia (2013), and Vesterinen et al. (2014), a sample size of 8 is widely accepted in studies of collagen-induced arthritis (CIA) and is adequate to identify significant differences in metrics such as arthritis and histopathological scores. This design provides a statistical power exceeding 80% for primary outcome measures.

#### 2.21.2 Gut microbiota analysis

Three groups were analyzed: control, model, and high-dose NBTL, with *n* = 8 per group. Gut microbiota composition and diversity were evaluated using 16S rDNA sequencing. Xu et al. (2020) and Liu J. et al. (2021) confirms that this sample size is sufficient to detect significant intergroup variations in microbiota profiles, enabling robust α and β diversity analyses, LEfSe analysis, and key taxa identification. The statistical power of the gut microbiota analysis was evaluated using R based on OTU-level data. The OTU table, metadata, and taxonomy data were merged using sample IDs, and the group variable (control, model, high-dose NBTL) was defined as an ordered factor. OTU abundance was normalized through *z*-score transformation to ensure consistency across samples. Significant OTUs were identified using Kruskal–Wallis tests (*p* < 0.05). Effect sizes were calculated for significant OTUs using MANOVA, and the average effect size was used for power analysis with the R “pwr” (Champely, 2020) package, assuming a sample size of *n* = 8 per group and three groups. The results indicated a statistical power of 0.9233 (Supplementary material S1), confirming the study’s sensitivity to detect significant differences in gut microbiota composition between groups.

#### 2.21.3 Synovial transcriptomics analysis

Groups included control, model, and high-dose NBTL, with each group consisting of six samples (*n* = 6). Synovial tissues were subjected to RNA-Seq analysis for gene expression and pathway enrichment. As noted by Love et al. (2014) and Shu et al. (2021), *n* = 6 is considered adequate for detecting differentially expressed genes (DEGs) and changes in pathway activity. Power analysis (Supplementary material S1) involved preprocessing count matrices, estimating dispersion using the “RnaSeqSampleSize” (Zhao et al., 2021) package, and calculating statistical power at a significance level of 0.01 and a fold-change threshold of 2. The power for transcriptomic data was calculated to be 0.8193.

#### 2.21.4 Deep neural network model prediction

Power analysis details (Supplementary material S2) include root-mean-square error (RMSE), Shapiro–Wilk tests for residual normality, and Pearson correlation coefficients. The model exhibited an RMSE of 0.084 (training) and 0.134 (testing), residual *p*-values of 0.124 (training) and 0.640 (testing), and Pearson correlation coefficients of 0.819 (training) and 0.979 (testing).

#### 2.21.5 Western blot

Six experimental groups—control, model, low-dose NBTL, medium-dose NBTL, high-dose NBTL, and positive control—were analyzed with *n* = 3 biological replicates per group. As per Chen et al. (2023), this sample size, combined with internal control normalization, suffices to detect significant differences in protein expression.

#### 2.21.6 Quantitative PCR

Six groups (control, model, low-dose NBTL, medium-dose NBTL, high-dose NBTL, and positive control) were assessed, with three biological replicates per group (*n* = 3) and three technical replicates per sample. According to Livak and Schmittgen (2001) and Nan et al. (2023), this configuration ensures stable and reliable results.

#### 2.21.7 ELISA

Groups included control, model, low-dose NBTL, medium-dose NBTL, high-dose NBTL, and positive control, with each group comprising three biological replicates (*n* = 3). As stated by Li et al. (2021), this sample size is adequate for measuring significant differences in cytokines such as TNF-α, IL-6, and IL-17.

#### 2.21.8 Cellular experiments

Two experimental groups were analyzed: control (20% blank serum) and NBTL-treated serum (20%), with three biological replicates per group (*n* = 3). This setup supported wound healing, tube formation, proliferation, and immunofluorescence assays. Referring to Qian et al. (2021) and Mosmann (1983), this sample size is commonly used in cellular studies and is sufficient to capture significant changes in angiogenesis, migration, and proliferation.

## 3 Results

### 3.1 Comprehensive analysis of prototype components and metabolites of NBTL in blood using UPHLC-MS-TOF

Using UPHLC-MS-TOF, we conducted an in-depth analysis of the prototype components and metabolites of new bitongling (NBTL) after its entry into the bloodstream.

**Figure 1:**
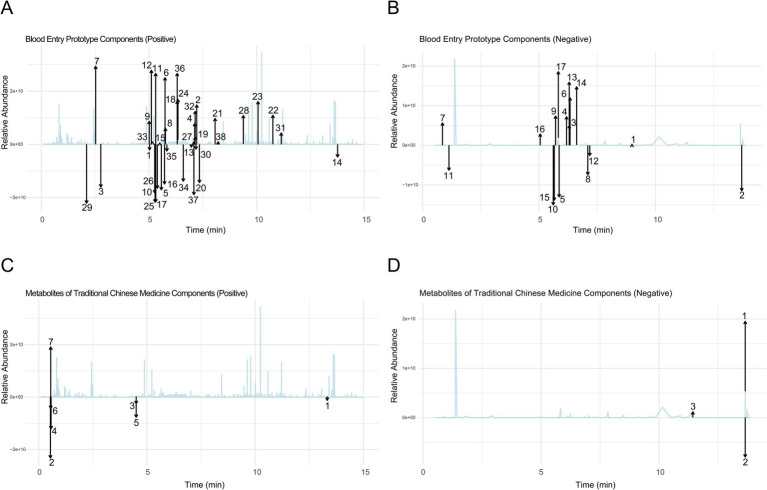
Comprehensive analysis of prototype components and metabolites of NBTL in blood using UPHLC-MS-TOF. **(A)** Blood entry prototype (positive). **(B)** Blood entry prototype (negative). **(C)** Metabolites of TCM (positive). **(D)** Metabolites of TCM (negative).

In the positive ion mode (Figure 1A and Table 3), the primary prototype components identified included zingerone (Rt. min. 5.029, *m*/*z*: 177.0907), wogonoside (Rt. min. 7.219, *m*/*z*: 461.1054), and uracil (Rt. min. 2.764, *m*/*z*: 113.0346), among others. In the negative ion mode (Figure 1B and Table 4), the main components detected were terpineol-4 (Rt. min. 8.989, *m*/*z*: 153.12506), succinic anhydride (Rt. min. 13.701, *m*/*z*: 99.00613), and salicyluric acid (Rt. min. 6.295, *m*/*z*: 194.04202), among others.

**Table 3:** Blood entry prototype components (positive).

Number	ESI mode	Rt. min.	*m*/*z*	Metabolite name	Adduct. type	Formula	INCHIKEY
1	Positive	5.029	177.0907	Zingerone	[M + H − H_2_O]^+^	C_11_H_14_O_3_	OJYLAHXKWMRDGS-UHFFFAOYSA-N
2	Positive	7.219	461.1054	Wogonoside	[M + H]^+^	C_22_H_20_O_11_	LNOHXHDWGCMVCO-NTKSAMNMSA-N
3	Positive	2.764	113.0346	Uracil	[M + H]^+^ B	C_4_H_4_N_2_O_2_	ISAKRJDGNUQOIC-UHFFFAOYSA-N
4	Positive	7.125	419.1689	Syringaresinol	[M + H]^+^	C_22_H_26_O_8_	KOWMJRJXZMEZLD-UHFFFAOYSA-N
5	Positive	5.575	298.1432	Stepharine	[M + H]^+^	C_18_H_19_NO_3_	OGJKMZVUJJYWKO-UHFFFAOYSA-N
6	Positive	5.746	323.1119	Salidroside	[M + Na]^+^	C_14_H_20_O_7_	ILRCGYURZSFMEG-RKQHYHRCSA-N
7	Positive	2.525	121.0647	Phenylacetaldehyde	[M + H]^+^	C_8_H_8_O	DTUQWGWMVIHBKE-UHFFFAOYSA-N
8	Positive	5.765	483.1474	Paeonolide	[M + Na]^+^	C_20_H_28_O_12_	IDZZECHGWAZTIB-NYBIBFQCSA-N
9	Positive	5.012	152.1068	Norephedrine	[M + H]^+^	C_9_H_13_NO	DLNKOYKMWOXYQA-VXNVDRBHSA-N
10	Positive	5.268	180.1377	N-Methylephedrine	[M + H]^+^	C_11_H_17_NO	FMCGSUUBYTWNDP-ONGXEEELSA-N
11	Positive	5.312	360.2523	Napelline	[M + H]^+^	C_22_H_33_NO_3_	AZAZKLKDEOMJBJ-UHFFFAOYSA-N
12	Positive	5.113	486.2679	Mesaconine	[M + H]^+^	C_24_H_39_NO_9_	GQRPJUIKGLHLLN-UHFFFAOYSA-N
13	Positive	6.969	301.1045	Meranzin hydrate	[M + Na]^+^	C_15_H_18_O_5_	KGGUASRIGLRPAX-UHFFFAOYSA-N
14	Positive	13.762	180.1015	Maltoxazine	[M + H − H_2_O]^+^	C_10_H_13_NO_2_	MTHASAHNRVFFOM-UHFFFAOYSA-N
15	Positive	5.503	247.1430	Lenticin	[M + H]^+^	C_14_H_18_N_2_O_2_	AOHCBEAZXHZMOR-UHFFFAOYSA-N
16	Positive	5.722	358.2005	Laudanosine	[M + H]^+^	C_21_H_27_NO_4_	KGPAYJZAMGEDIQ-KRWDZBQOSA-N
17	Positive	5.283	408.2725	Isotalatizidine	[M + H]^+^	C_23_H_37_NO_5_	RBSZCNOWHDHRFZ-CFIIAAHPSA-N
18	Positive	6.315	162.0907	Indole-3-ethanol	[M + H]^+^	C_10_H_11_NO	MBBOMCVGYCRMEA-UHFFFAOYSA-N
19	Positive	7.146	466.3147	Glycocholic acid	[M + H]^+^	C_26_H_43_NO_6_	RFDAIACWWDREDC-FRVQLJSFSA-N
20	Positive	7.344	169.1220	Geranic acid	[M + H]^+^	C_10_H_16_O_2_	ZHYZQXUYZJNEHD-UHFFFAOYSA-N
21	Positive	8.071	269.0803	Formononetin	[M + H]^+^	C_16_H_12_O_4_	HKQYGTCOTHHOMP-UHFFFAOYSA-N
22	Positive	10.755	338.3408	Erucamide	[M + H]^+^	C_22_H_43_NO	UAUDZVJPLUQNMU-KTKRTIGZSA-N
23	Positive	10.067	297.1471	Cryptotanshinone	[M + H]^+^	C_19_H_20_O_3_	GVKKJJOMQCNPGB-JTQLQIEISA-N
24	Positive	6.347	307.1159	Cimifugin	[M + H]^+^	C_16_H_18_O_6_	ATDBDSBKYKMRGZ-UHFFFAOYSA-N
25	Positive	5.317	316.1526	Cephalotaxine	[M + H]^+^	C_18_H_21_NO_4_	YMNCVRSYJBNGLD-KZNAEPCWSA-N
26	Positive	5.389	358.2360	Bullatine G	[M + H]^+^	C_22_H_31_NO_3_	CBOSLVQFGANWTL-JAQKJRLSSA-N
27	Positive	7.087	293.1004	Benzyl glucopyranoside	[M + Na]^+^	C_13_H_18_O_6_	GKHCBYYBLTXYEV-UJPOAAIJSA-N
28	Positive	9.384	308.0906	Arborinine	[M + Na]^+^	C_16_H_15_NO_4_	ATBZZQPALSPNMF-UHFFFAOYSA-N
29	Positive	2.104	158.0921	Amphetamine	[M + Na]^+^	C_9_H_13_N	KWTSXDURSIMDCE-QMMMGPOBSA-N
30	Positive	7.183	247.1319	Abscisate	[M + H − H_2_O]^+^	C_15_H_20_O_4_	JLIDBLDQVAYHNE-WEYXYWBQSA-N
31	Positive	11.143	419.1325	5-Hydroxy-6,7,8,3′,4′,5′-hexamethoxyflavone	[M + H]^+^	C_21_H_22_O_9_	MQBFFYQCZCKSBX-UHFFFAOYSA-N
32	Positive	7.146	313.1035	5,7-Dimethoxy-2-(4-methoxyphenyl)-4H-chromen-4-one	[M + H]^+^	C_18_H_16_O_5_	ZXJJBDHPUHUUHD-UHFFFAOYSA-N
33	Positive	5.156	148.1114	5,6,7,8-Tetrahydro-4-methylquinoline	[M + CH_3_OH + H]^+^	C_10_H_13_N	LGYCOYCCCKHXGC-UHFFFAOYSA-N
34	Positive	6.594	135.0436	4-Methoxybenzoate	[M + H − H_2_O]^+^	C_8_H_8_O_3_	ZEYHEAKUIGZSGI-UHFFFAOYSA-N
35	Positive	5.832	220.0594	3,4-Dihydroxy-L-phenylalanine	[M + Na]^+^	C_9_H_11_NO_4_	WTDRDQBEARUVNC-LURJTMIESA-N
36	Positive	6.312	137.0593	2-Hydroxyacetophenone	[M + H]^+^	C_8_H_8_O_2_	ZWVHTXAYIKBMEE-UHFFFAOYSA-N
37	Positive	7.087	461.1053	(2S,3S,4S,5R,6S)-3,4,5-trihydroxy-6-(5-hydroxy-8-methoxy-4-Oxo-2-phenylchromen-7-Yl)oxyoxane-2-carboxylic acid	[M + H]^+^	C_22_H_20_O_11_	LNOHXHDWGCMVCO-NTKSAMNMSA-N
38	Positive	8.213	277.1058	(2S)-4-hydroxy-2-(2-hydroxypropan-2-Yl)-7-methyl-2,3-dihydrofuro[3,2-G]chromen-5-one	[M + H]^+^	C_15_H_16_O_5_	LJSWMDKKEBOERP-UHFFFAOYSA-N

**Table 4:** Blood entry prototype components (negative).

Number	ESI mode	Rt. min.	*m*/*z*	Metabolite name	Adduct. type	Formula	INCHIKEY
1	Negative	8.989	153.12506	Terpineol-4	[M − H]^−^	C_10_H_18_O	WRYLYDPHFGVWKC-UHFFFAOYSA-N
2	Negative	13.701	99.00613	Succinic anhydride	[M − H_2_O − H]^−^	C_4_H_4_O_3_	RINCXYDBBGOEEQ-UHFFFAOYSA-N
3	Negative	6.295	194.04202	Salicyluric acid	[M − H]^−^	C_9_H_9_NO_4_	ONJSZLXSECQROL-UHFFFAOYSA-N
4	Negative	6.179	463.08154	Quercetin 3-O-glucoside	[M − H]^−^	C_21_H_20_O_12_	OVSQVDMCBVZWGM-QSOFNFLRSA-N
5	Negative	5.861	192.06271	Phenylacetylglycine	[M − H]^−^	C_10_H_11_NO_3_	UTYVDVLMYQPLQB-UHFFFAOYSA-N
6	Negative	6.330	579.16412	Naringin	[M − H]^−^	C_27_H_32_O_14_	DFPMSGMNTNDNHN-UHFFFAOYSA-N
7	Negative	0.853	245.03946	Glycerophosphoglycerol	[M−H]−	C_6_H_15_O_8_P	LLCSXHMJULHSJN-OLQVQODUSA-N
8	Negative	7.092	183.06276	Genipic acid	[M − H]^−^	C_9_H_12_O_4_	KWBASGHXHPTPGU-UHFFFAOYSA-N
9	Negative	5.708	787.25836	Eleutheroside E	[M + FA − H]^−^	C_34_H_46_O_18_	FFDULTAFAQRACT-XKBSQSBASA-N
10	Negative	5.609	415.09717	Daidzein-8-C-glucoside	[M − H]^−^	C_21_H_20_O_9_	HKEAFJYKMMKDOR-UHFFFAOYSA-N
11	Negative	1.131	133.01071	D-(+)-malic acid	[M − H]^−^	C_4_H_6_O_5_	BJEPYKJPYRNKOW-REOHCLBHSA-N
12	Negative	7.178	263.12500	Abscisic acid; LC-tDDA	[M − H]^−^	C_15_H_20_O_4_	JLIDBLDQVAYHNE-WEYXYWBQSA-N
13	Negative	6.295	515.11108	3,4-Di-O-caffeoylquinic acid	[M − H]^−^	C_25_H_24_O_12_	UFCLZKMFXSILNL-RVXRWRFUSA-N
14	Negative	6.613	165.05206	3-(2-Hydroxyphenyl)propionic acid	[M − H]^−^	C_9_H_10_O_3_	CJBDUOMQLFKVQC-UHFFFAOYSA-N
15	Negative	5.653	153.01595	2,5-Dihydroxybenzoate	[M − H]^−^	C_7_H_6_O_4_	WXTMDXOMEHJXQO-UHFFFAOYSA-N
16	Negative	5.049	255.04716	2,3-Dihydroxy-2-[(4-hydroxyphenyl)methyl]butanedioic acid	[M − H]^−^	C_11_H_12_O_7_	TUODPMGCCJSJRH-KWQFWETISA-N
17	Negative	5.827	211.99826	1H-Indol-3-yloxidanesulfonic acid	[M − H]^−^	C_8_H_7_NO_4_S	BXFFHSIDQOFMLE-UHFFFAOYSA-N

Furthermore, the analysis of NBTL metabolites in the bloodstream revealed several key compounds. In the positive ion mode (Figure 1C and Table 5), we identified pyrimidine (Rt. min. 13.328, *m*/*z*: 81.04522), pyridine (Rt. min. 0.539, *m*/*z*: 80.04992), and myrcene (Rt. min. 4.501, *m*/*z*: 137.13211), among others. In the negative ion mode (Figure 1D and Table 6), the primary metabolites detected included D-galactose (Rt. min. 13.649, *m*/*z*: 179.0527), arabitol(D) (Rt. min. 13.649, *m*/*z*: 151.0578), and (+)-triptophenolide (Rt. min. 11.431, *m*/*z*: 311.1644), among others.

**Table 5:** Metabolites of traditional Chinese medicine components (positive).

Number	ESI mode	Rt. min.	*m*/*z*	Metabolite name	Adduct. type	Formula	INCHIKEY
1	Positive	13.328	81.04522	Pyrimidine	[M + H]^+^	C_4_H_4_N_2_	CZPWVGJYEJSRLH-UHFFFAOYSA-N
2	Positive	0.539	80.04992	Pyridine	[M + H]^+^	C_5_H_5_N	JUJWROOIHBZHMG-UHFFFAOYSA-N
3	Positive	4.501	137.13211	Myrcene	[M + H]^+^	C_10_H_16_	UAHWPYUMFXYFJY-UHFFFAOYSA-N
4	Positive	0.568	88.07614	Morpholine	[M + H]^+^	C_4_H_9_NO	YNAVUWVOSKDBBP-UHFFFAOYSA-N
5	Positive	4.501	153.12703	Camphora	[M + H]^+^	C_10_H_16_O	DSSYKIVIOFKYAU-XCBNKYQSSA-N
6	Positive	0.554	127.03881	1,2,3-Trihydroxybenzene	[M + H]^+^	C_6_H_6_O_3_	WQGWDDDVZFFDIG-UHFFFAOYSA-N
7	Positive	0.554	277.17914	[6]-Gingerol	[M + H − H_2_O]^+^	C_17_H_26_O_4_	NLDDIKRKFXEWBK-UHFFFAOYSA-N

**Table 6:** Metabolites of traditional Chinese medicine components (negative).

Number	ESI mode	Rt. min.	*m*/*z*	Metabolite name	Adduct. type	Formula	INCHIKEY
1	Negative	13.649	179.0527	D-Galactose	[M − H]^−^	C_6_H_12_O_6_	WQZGKKKJIJFFOK-PHYPRBDBSA-N
2	Negative	13.649	151.0578	Arabitol(D)	[M − H]^−^	C_5_H_12_O_5_	HEBKCHPVOIAQTA-IMJSIDKUSA-N
3	Negative	11.431	311.1644	(+)-Triptophenolide	[M − H]^−^	C_20_H_24_O_3_	KPXIBWGPZSPABK-FXAWDEMLSA-N

This detailed UPHLC-MS-TOF analysis successfully identified the prototype components and metabolites of NBTL in the bloodstream, providing crucial data to support further research into the pharmacological mechanisms of NBTL.

### 3.2 Assessment of NBTL efficacy in a rat arthritis model

**Figure 2:**
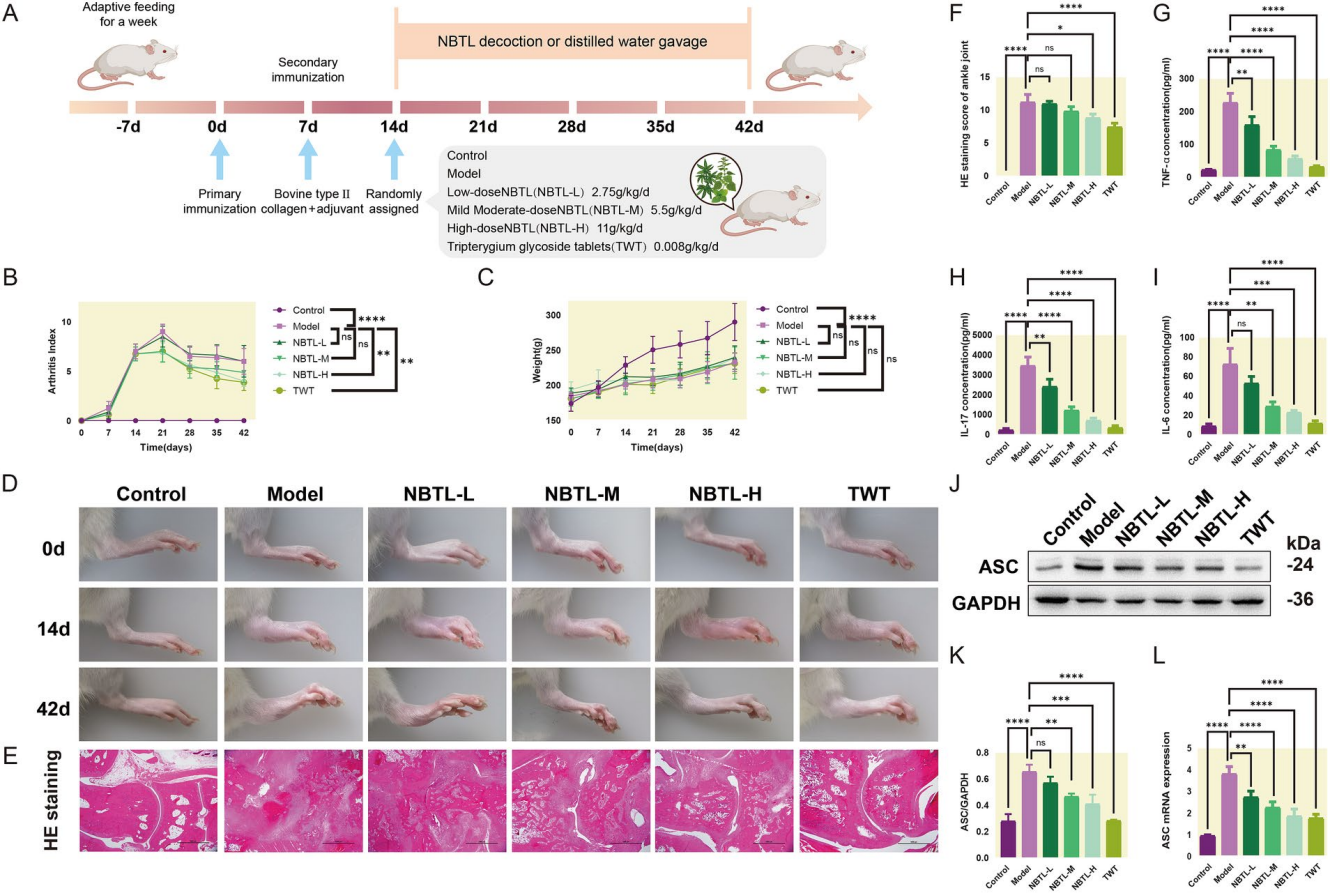
Evaluation of the therapeutic effects of NBTL on a rat model of arthritis. **(A)** Experimental design and timeline: −7 days to 0 day, acclimatization; 0 day, first immunization with type II collagen and adjuvant; 7 days, second immunization to enhance arthritis model; 14 days to 42 days, treatment groups: control (no treatment), model (arthritis development), new bitongling (NBTL) low (NBTL-L), medium (NBTL-M), and high (NBTL-H) doses, and Tripterygium wilfordii tablets (TWT) as a drug control. **(B)** Arthritis index scoring over time. **(C)** Weight changes, showing no significant differences (ns). **(D)** Visual comparison of rat leg inflammation on days 0, 14, and 42 across all groups. **(E)** Histological analysis with HE staining at 20× magnification showing joint tissue structure and inflammation. **(F)** HE staining scores indicating tissue damage severity. **(G,H)** Serum levels of TNF-α and IL-17, showing inflammation response across treatments. **(I)** IL-6 serum levels with significant reductions in treated groups compared to the model. **(J,K)** Western blot analysis of ASC protein expression in synovial tissue with GAPDH as a loading control. **(L)** ASC mRNA levels quantified by qPCR, comparing each group to the model, with statistical significance noted. Significance markers used are (^*^p < 0.05, ^**^p < 0.01, ^***^p < 0.001, and ^****^p < 0.0001, ns: not significant). **(B–F)** Sample size per group: *n* = 8. **(G–L)** Sample size per group: *n* = 3.

The animals underwent a one-week acclimation period to adjust to the experimental environment. Following this, two immunizations were performed on day 0 and day 7 to establish an arthritis model. The experimental animals were randomly divided into six groups: control, model, low-dose new bitongling (NBTL-L), medium-dose new bitongling (NBTL-M), high-dose new bitongling (NBTL-H), and Tripterygium wilfordii tablets (TWT). Treatment for each group began on day 14 and continued until day 42 (Figure 2A).

Compared to the model group, all new bitongling groups and the TWT group showed reductions in arthritis index, especially the NBTL-H group (*p* < 0.01) (Figure 2B). Body weight changes during the experiment did not differ significantly across the groups (Figure 2C). Photographs taken at various time points revealed typical arthritis symptoms in the model group, which were noticeably reduced in the new bitongling and TWT groups (Figure 2D).

Histopathological examination of the ankle joints in the model group revealed significant structural damage along with marked infiltration of inflammatory cells. In contrast, both the NBTL-H and TWT groups showed a notable reduction in these pathological changes (*p* < 0.05 and *p* < 0.0001) compared to the model group (Figures 2E,F).

Following new bitongling treatment, TNF-α and IL-17 levels were notably decreased in the NBTL-L, NBTL-M, and NBTL-H groups (*p* < 0.01 and *p* < 0.0001), with comparable reductions seen in the TWT group (Figures 2G,H). Regarding IL-6, significant reductions were noted in the NBTL-M and NBTL-H groups (*p* < 0.01 and *p* < 0.001), but not in the NBTL-L group (Figure 2I).

Western blot analysis showed significantly increased ASC protein expression in the synovial tissues of the model group. After new bitongling treatment, ASC expression showed a dose-dependent decline, with the greatest reduction observed in the NBTL-H group (*p* < 0.001). The TWT group also showed a similar decreasing trend (Figures 2J,K). Quantitative polymerase chain reaction (qPCR) results further confirmed that ASC mRNA levels in the synovial tissues were significantly reduced in the NBTL-H, and TWT groups (*p* < 0.0001) (Figure 2L).

### 3.3 Gut microbiota structure and diversity analysis under NBTL-H treatment

**Figure 3:**
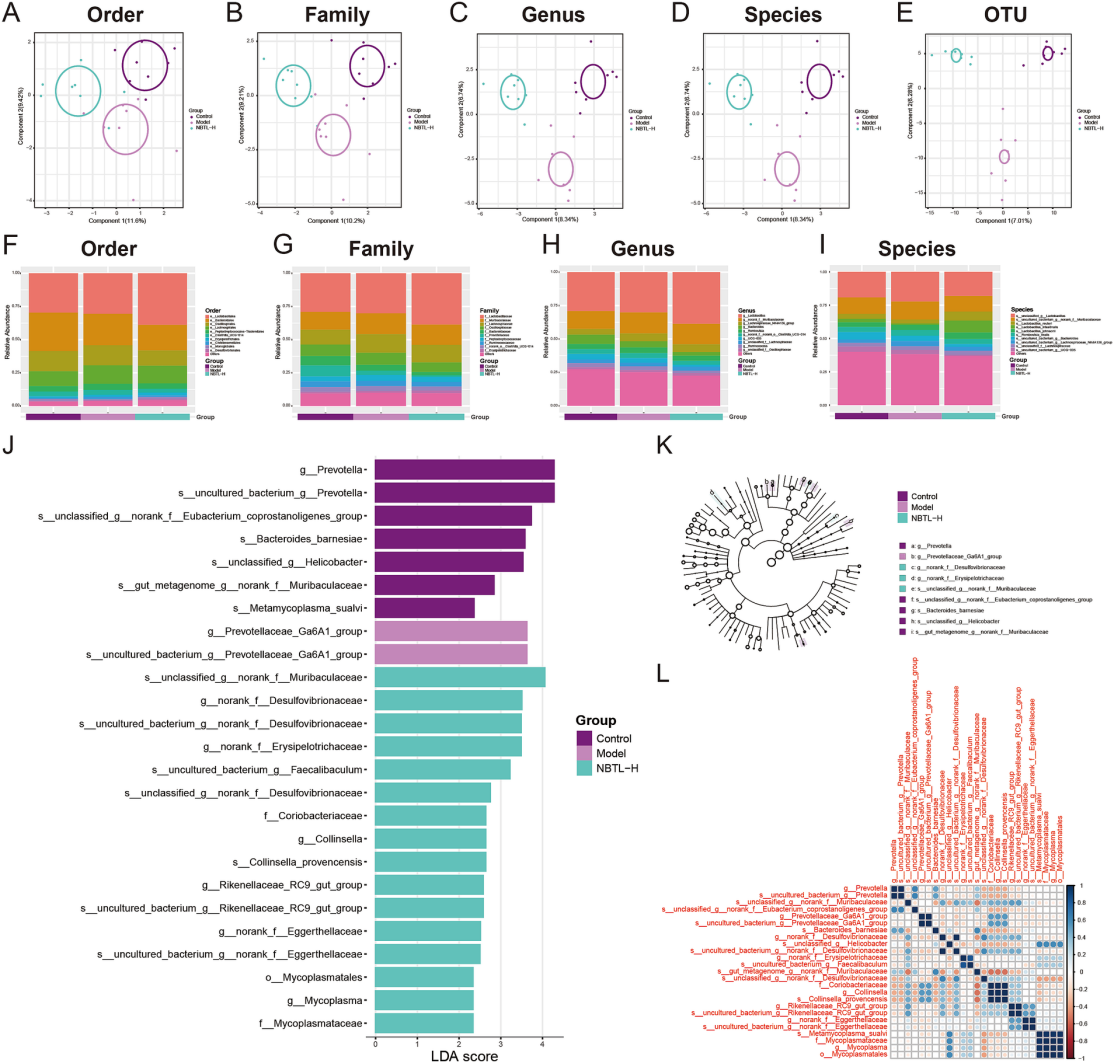
Gut microbiota structure and diversity analysis under NBTL-H treatment. **(A)** Order level: PLS-DA analysis shows 11.6% of the variance explained by the first principal component and 9.42% by the second. Different groups are marked with distinct colors: purple for control, red for model, and green for NBTL-H (high dose of new bitongling). **(B)** Family level: 10.2% of the variance is captured by the first principal component and 9.21% by the second, with consistent colors and markers. **(C)** Genus level: the first and second principal components account for 8.34 and 6.74% of the variance, respectively, with colors and markers as in the order level. **(D)** Species level: both the first and second components explain 8.34 and 6.74% of the variance, respectively, maintaining the same color scheme. **(E)** OTU level: 7.01% of the variance is explained by the first component and 6.28% by the second, using the same colors and markers as in the order level. **(F–I)** Depicts the relative abundance of gut microbiota in the control, model, and high-dose new bitongling (NBTL-H) groups, analyzed at the order **(F)**, family **(G)**, genus **(H)**, and species **(I)** levels. Bar charts are used for each category, with varying colors representing different taxa. **(J)** LDA score plot: this plot highlights microbial taxa with significant differences across the control, model, and NBTL-H groups, with each bar’s length reflecting the LDA score. **(K)** Phylogenetic tree: significant differences in microbial taxa between groups based on LDA scores, with branch end circles sized proportionally to significance and colors indicating different groups. **(L)** Correlation heatmap: correlations of significantly different microbial taxa in the three groups, with color intensity indicating correlation strength, red for positive correlations, and blue for negative correlations. **(A–L)** Sample size per group: *n* = 8.

In this study, we employed PLS-DA to deeply analyze the gut microbiota structure across various taxonomic levels: order (Figure 3A), family (Figure 3B), genus (Figure 3C), species (Figure 3D), and operational taxonomic units (OTU) (Figure 3E). Experimental data points are marked in purple, red, and green to represent the control group (control), model group (model), and high-dose new bitongling treatment group (NBTL-H), respectively. Each subplot reveals the main directions of data variation through the first and second principal components, with the corresponding variance percentages labeled on the axes. The principal component analysis results clearly demonstrate the distribution characteristics of the three groups within the principal component space, with the NBTL-H treatment group showing significant separation at multiple taxonomic levels.

We also comprehensively analyzed the microbial abundance distribution at the order (Figure 3F), family (Figure 3G), genus (Figure 3H), and species (Figure 3I) levels. The data is displayed using distinctly colored bar charts, highlighting the abundance of each microbial group. The control, model, and NBTL-H groups are marked by different color bands at the chart base, aiding in the comparative analysis of gut microbiota across various taxonomic levels. Notable differences were observed in the proportions of certain microbial groups. For instance, at the order level (Figure 3F), the abundance of *Bacteroidales* decreased in the NBTL-H treatment group compared to the control and model groups. At the family level (Figure 3G), *Prevotellaceae* abundance decreased in the treatment group relative to the model group. At the genus level (Figure 3H), *UCG-005* abundance significantly declined in the high-dose treatment group. At the species level (Figure 3I), *Lactobacillus johnsonii* showed increased abundance in the high-dose treatment group relative to the other two groups.

Additionally, we applied LEfSe to detect significant microbial taxa differences among the control, model, and NBTL-H groups. The linear discriminant analysis (LDA) scores indicated the degree of intergroup differences, with higher scores reflecting greater differences. Analysis revealed that *f_Mycoplasmataceae*, *g_Prevotellaceae_Ga6A1_group*, *s_Metamycoplasma_sualvi*, *s_uncultured_bacterium_g_norank_f_Eggerthellaceae*, and *s_uncultured_bacterium_g_Prevotellaceae_Ga6A1_group* exhibited high LDA scores, indicating significant abundance differences among groups (Figure 3J). Furthermore, the phylogenetic tree generated based on LDA scores (Figure 3K) illustrated the evolutionary relationships of these significantly different microbial taxa. Each node’s size represents the significance of the corresponding taxon, and color differences reflect different experimental groups.

Correlation heatmap analysis (Figure 3L) revealed significant correlations of *f_Mycoplasmataceae* with various other microbial taxa, especially with *g_Prevotellaceae_Ga6A1_group* in the NBTL-H treatment group. Additionally, changes in the correlation patterns of *s_Metamycoplasma_sualvi* with *s_uncultured_bacterium_g_norank_f_Eggerthellaceae* and *s_uncultured_bacterium_g_Prevotellaceae_Ga6A1_group* might reflect ecological interactions or similar physiological metabolic pathways. Notably, the altered correlations of *s_Metamycoplasma_sualvi* post-treatment suggest that NBTL may impact specific gut microbial interactions. Furthermore, significant correlation changes for *s_uncultured_bacterium_g_Prevotellaceae_Ga6A1_group* indicate that this uncultured taxon may undergo niche alterations under drug influence.

### 3.4 Identifying characteristic gut microbiota for NBTL through machine learning

**Figure 4:**
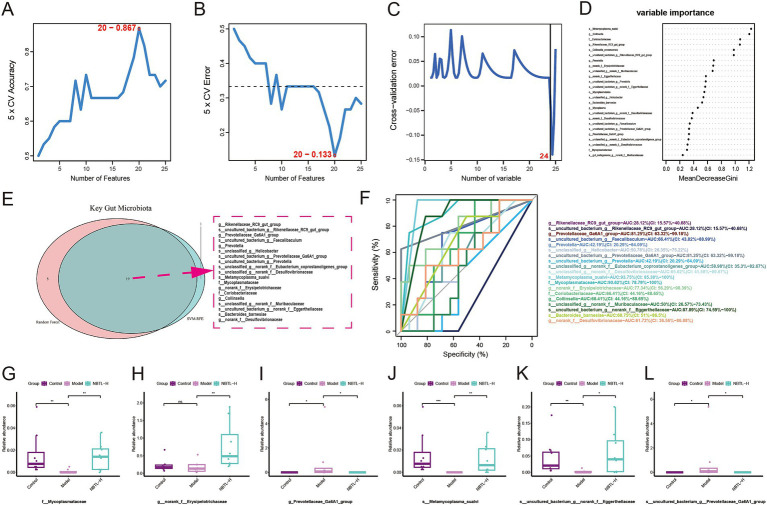
Identifying characteristic gut microbiota for NBTL through machine learning. **(A)** In the SVM-RFE method, the accuracy of 5-fold cross-validation varies with the number of features, reaching a maximum of 0.867 with 20 features. **(B)** The error rate in 5-fold cross-validation using the SVM-RFE method decreases with the number of features, reaching a minimum of 0.133 with 20 features. **(C)** In the random forest method, the cross-validation error rate varies with the number of variables, with the lowest error at 24 variables. **(D)** Variable importance in the random forest model is represented by MeanDecreaseGini. **(E)** Venn diagram of key gut microbiota identified by SVM-RFE and random forest methods, with 19 common taxa highlighted by a red dashed border. **(F)** ROC curves of key taxa show the relationship between sensitivity and specificity at different thresholds, with different colors representing different taxa and corresponding AUC values listed in the legend. **(G–L)** Box plots illustrating the relative abundance of *f_Mycoplasmataceae* (G), *g_norank_f_Erysipelotrichaceae* **(H)**, *g_Prevotellaceae_Ga6A1_group* **(I)**, *s_Metamycoplasma_sualvi* **(J)**, *s_uncultured_bacterium_g_norank_f_Eggerthellaceae* **(K)**, and *s_uncultured_bacterium_g_Prevotellaceae_Ga6A1_group* **(L)** across the control, model, and NBTL-H groups. ^*^p < 0.05, ^**^p < 0.01, and ^***^p < 0.001, ns: not significant. **(A–L)** Sample size per group: *n* = 8.

The SVM-RFE analysis results demonstrate a gradual improvement in the model’s 5-fold cross-validation accuracy (Figure 4A), which reaches its peak at 0.867 with 20 features. Simultaneously, the 5-fold cross-validation error (Figure 4B) decreases to its lowest point of 0.133 at 20 features, indicating optimal model performance at this point. The random forest analysis results show a steady decline in cross-validation error (Figure 4C) as the number of variables increases, reaching its lowest at 24 variables, demonstrating the model’s excellent generalization ability. The variable importance assessment (Figure 4D) highlights significant contributions to the model’s predictive power from species such as *s_Metamycoplasma_sualvi* and *s_uncultured_bacterium_g_norank_f_Eggerthellaceae*. The Venn diagram (Figure 4E) reveals a consensus between SVM-RFE and random forest in identifying 19 key microbial taxa, including *f_Mycoplasmataceae* and *g_Prevotellaceae_Ga6A1_group*, as potential targets for new bitongling’s regulation of the gut microbiome.

For the 19 key microbial taxa identified by machine learning, ROC analysis assessed their sensitivity and specificity across various classification thresholds. Six taxa had area under the curve (AUC) values exceeding 70%, indicating strong discriminative power. These include *g_Prevotellaceae_Ga6A1_group* and *s_uncultured_bacterium_g_Prevotellaceae_Ga6A1_group* with AUCs of 81.25%, *s_Metamycoplasma_sualvi* with an AUC of 93.75%, *f_Mycoplasmataceae* with an AUC of 90.62%, *g_norank_f_Erysipelotrichaceae* with an AUC of 77.34%, and *s_uncultured_bacterium_g_norank_f_Eggerthellaceae* with an AUC of 87.89% (Figure 4F).

Box plots illustrate the relative abundance differences of these taxa among the control, model, and NBTL-H. In the model condition, the relative abundance of *g_Prevotellaceae_Ga6A1_group* (Figure 4I) and *s_uncultured_bacterium_g_Prevotellaceae_Ga6A1_group* (Figure 4L) was markedly higher than in the control group but decreased significantly following NBTL-H treatment. In contrast, the abundance of *f_Mycoplasmataceae* (Figure 4G), *s_Metamycoplasma_sualvi* (Figure 4J), and *s_uncultured_bacterium_g_norank_f_Eggerthellaceae* (Figure 4K) was notably lower in the model group, yet it significantly increased after NBTL-H treatment. However, *g_norank_f_Erysipelotrichaceae* (Figure 4H) showed significant differences between the NBTL-H treatment and model groups, but not between the control and model groups, resulting in its exclusion.

Considering the high discriminative power of the ROC curves and the significant differences among the three groups, we identified five taxa as characteristic microbial taxa influenced by NBTL: *f_Mycoplasmataceae*, *g_Prevotellaceae_Ga6A1_group*, *s_uncultured_bacterium_g_Prevotellaceae_Ga6A1_group*, *s_Metamycoplasma_sualvi*, and *s_uncultured_bacterium_g_norank_f_Eggerthellaceae*.

### 3.5 Transcriptome analysis reveals NBTL’s impact on VEGF angiogenesis pathway and correlation of VEGF mRNA expression with characteristic gut microbiota

**Figure 5:**
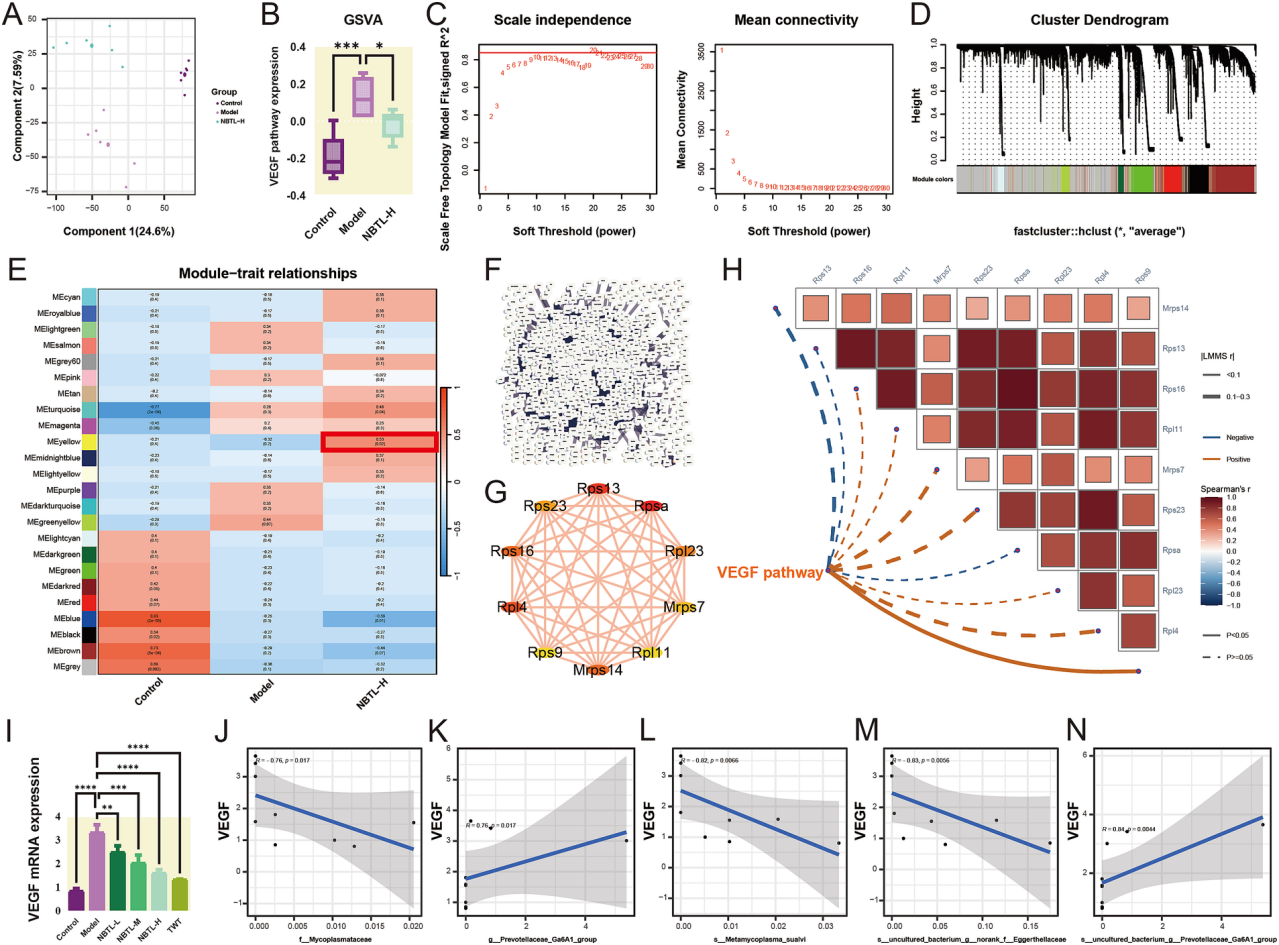
Transcriptome analysis reveals NBTL’s impact on VEGF angiogenesis pathway and correlation of VEGF mRNA expression with characteristic gut microbiota. **(A)** PLS-DA scatter plot with PC1 (24.6% variance) and PC2 (7.59% variance). Purple, red, and green dots represent control, model, and NBTL-H groups, respectively. **(B)** VEGF pathway box plot showing distribution, median, quartiles, and outliers. Purple, red, and green boxes represent control, model, and NBTL-H groups, respectively. ^*^p < 0.05 and ^***^p < 0.001. **(C)** Scale independence and mean connectivity vs. soft threshold for network construction. **(D)** Gene module cluster dendrogram with modules colored. **(E)** Module-trait relationship heatmap depicting module-sample correlations: red signifies positive, blue indicates negative; numbers represent the correlation coefficients and *p*-values. Highlighted modules are significantly associated with NBTL-H. **(F)** PPI network for NBTL-H module genes. **(G)** Top 10 genes by degree in the NBTL-H module, with node size representing degree and edge thickness interaction strength. **(H)** GSVA VEGF pathway data with Spearman correlations for the top 10 genes by degree, using solid lines for *p* < 0.05 and dashed lines for *p* > 0.05. Positive correlations are shown in red, while negative correlations are in blue. **(I)** VEGF mRNA expression comparison across control, model, NBTL-L, NBTL-M, NBTL-H, and TWT groups. ^**^p < 0.01, ^***^p < 0.001, and ^****^p < 0.0001. **(J–N)** Spearman correlation between VEGF mRNA and bacterial taxa in NBTL-H rats: **(J)** *f_Mycoplasmataceae* (*r* = −0.76, *p* = 0.017), **(K)** *g_Prevotellaceae_Ga6A1_group* (*r* = 0.76, *p* = 0.017), **(L)** *s_Metamycoplasma_sualvi* (*r* = −0.82, *p* = 0.0066), **(M)** *s_uncultured_bacterium_g_norank_f_Eggerthellaceae* (*r* = −0.83, *p* = 0.0056), **(N)** *s_uncultured_bacterium_g_Prevotellaceae_Ga6A1_group* (*r* = 0.84, *p* = 0.0044). Gray areas represent the 95% confidence interval, with significance marked at *p* < 0.05. **(A–H)** Sample size per group: *n* = 6. **(I–N)** Sample size per group: *n* = 3.

Transcriptome analysis results (Figure 5A) through PLS-DA show that PC explain 24.6 and 7.59% of the total variation, respectively, clearly distinguishing between the control, model, and NBTL-H groups. gene set variation analysis (GSVA) (Figure 5B) indicates a significant increase in the VEGF signaling pathway in the model group compared to the control group (*p* < 0.001), while a significant downregulation is observed in the NBTL-H group compared to the model group (*p* < 0.05).

Further investigation into the impact of NBTL-H on gene expression patterns was conducted using WGCNA. The soft threshold power analysis (Figure 5C) identified the optimal threshold as 20, optimizing the scale-free topology of the network. In the gene clustering dendrogram (Figure 5D), different modules were distinguished by color. The module-trait relationship heatmap (Figure 5E) revealed that the MEyellow module had a significant positive correlation with the NBTL-H group (*r* = 0.53, *p* = 0.02), suggesting that NBTL affects the VEGF pathway by regulating genes within this module, which is crucial for understanding the drug’s mechanism.

We also constructed a PPI network for the NBTL-H module genes (Figure 5F), revealing complex interactions among the drug module genes. Using Cytoscape, we visualized the top 10 genes with the highest degrees within the PPI network (Figure 5G), indicating their potential key roles in the mechanism of NBTL. Spearman analysis (Figure 5H) revealed a strong positive association between Rps9 and VEGF pathway activity, indicating a potential involvement of Rps9 in NBTL’s mechanism via the VEGF pathway. Although other genes (such as Rps16, Rpl11, etc.) did not show significant correlations with the VEGF pathway (*p* > 0.05), they might still be involved in the pathway through more complex mechanisms.

The study also assessed the effects of different doses of NBTL on VEGF mRNA expression (Figure 5I). VEGF mRNA levels were markedly elevated in the model group, whereas all NBTL doses, particularly the high dose, led to a notable reduction. The positive control group treated with Tripterygium wilfordii tablets (TWT) also showed a similar reduction. Additionally, Spearman correlation analysis revealed significant associations between NBTL-H treatment and characteristic gut microbiota. Specifically, the abundance of *f_Mycoplasmataceae* (Figure 5J, *r* = −0.76, *p* = 0.017), *s_Metamycoplasma_sualvi* (Figure 5L, *r* = −0.82, *p* = 0.0066), and *s_uncultured_bacterium_g_norank_f_Eggerthellaceae* (Figure 5M, *r* = −0.83, *p* = 0.0056) showed a negative correlation with VEGF mRNA expression, while *g_Prevotellaceae_Ga6A1_group* (Figure 5K, *r* = 0.76, *p* = 0.017) and *s_uncultured_bacterium_g_Prevotellaceae_Ga6A1_group* (Figure 5N, *r* = 0.84, *p* = 0.0044) showed a positive correlation.

### 3.6 Dose-dependent regulation of miR-20a-5p and miR-223-3p by NBTL and their correlation with VEGF expression and characteristic gut microbiota

**Figure 6:**
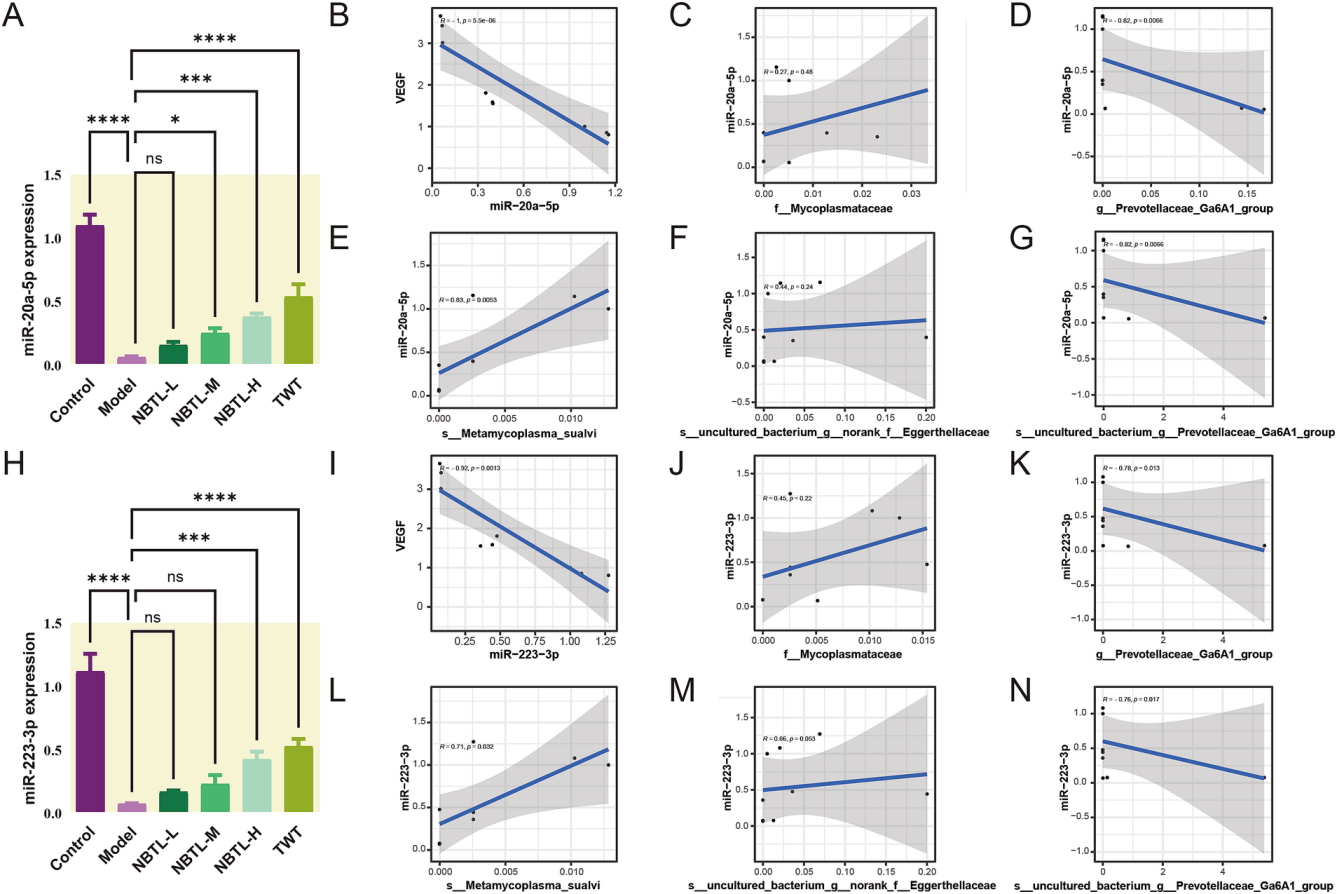
Dose-dependent regulation of miR-20a-5p and miR-223-3p by NBTL and their correlation with VEGF expression and characteristic gut microbiota. **(A)** Comparison of miR-20a-5p expression levels across different treatment groups in rats. **(B)** Spearman correlation analysis between miR-20a-5p and VEGF mRNA expression in the NBTL-H group showed a significant negative correlation (*r* = −1, *p* = 5.6 × 10^−6^). **(C)** The abundance of the *f_Mycoplasmataceae* family exhibited a low positive correlation with miR-20a-5p, though not statistically significant (*r* = 0.27, *p* = 0.48). **(D)** The abundance of *g_Prevotellaceae_Ga6A1_group* showed a strong negative correlation with miR-20a-5p (*r* = −0.82, *p* = 0.0066). **(E)** The abundance of *s_Metamycoplasma_sualvi* species showed a positive correlation with miR-20a-5p (*r* = 0.83, *p* = 0.0053). **(F)** The *s_uncultured_bacterium_g_norank_f_Eggerthellaceae* species exhibited a low positive correlation with miR-20a-5p, not reaching statistical significance (*r* = 0.44, *p* = 0.24). **(G)** The species *s_uncultured_bacterium_g*_*Prevotellaceae_Ga6A1_group* was strongly negatively correlated with miR-20a-5p (*r* = −0.82, *p* = 0.0066). **(H)** Comparison of miR-223-3p expression levels across different treatment groups in rats. **(I)** Spearman correlation analysis between miR-223-3p and VEGF mRNA expression in the NBTL-H group showed a negative correlation (*r* = −0.92, *p* = 0.0013). **(J)** The abundance of the *f_Mycoplasmataceae* family exhibited a low positive correlation with miR-223-3p, not reaching statistical significance (*r* = 0.45, *p* = 0.22). **(K)** The *g_Prevotellaceae_Ga6A1_group* genus abundance showed a negative correlation with miR-223-3p (*r* = −0.78, *p* = 0.013). **(L)** The abundance of *s_Metamycoplasma_sualvi* species demonstrated a positive correlation with miR-223-3p (*r* = 0.71, *p* = 0.032). **(M)** The *s_uncultured_bacterium_g_norank_f_Eggerthellaceae* species showed a low positive correlation with miR-223-3p, nearly reaching statistical significance (*r* = 0.66, *p* = 0.053). **(N)** The *s_uncultured_bacterium_g_Prevotellaceae_Ga6A1_group* species exhibited a negative correlation with miR-223-3p (*r* = −0.76, *p* = 0.017). The gray areas represent the confidence intervals, with significance at *p* < 0.05. ^*^p < 0.05, ^***^p < 0.001, and ^****^p < 0.0001; ns denotes no statistical significance. **(A–N)** Sample size per group: *n* = 3.

Our findings (Figures6A,H) revealed that in the model group, the levels of miR-20a-5p and miR-223-3p were significantly reduced, likely contributing to increased angiogenesis in the disease state. Treatment with NBTL, especially at high doses (NBTL-H), significantly restored the expression levels of these miRNAs. Likewise, expression recovery was observed in the positive control group receiving tripterygium glycosides, showing a similar trend. Spearman correlation analysis further demonstrated that, in the high-dose NBTL group, miR-20a-5p and miR-223-3p were significantly negatively correlated with VEGF mRNA expression (Figure 6B, *r* = −1, *p* = 5.6 × 10^−6^; Figure 6I, *r* = −0.92, *p* = 0.0013), suggesting these miRNAs may inhibit VEGF expression and, consequently, angiogenesis in the disease context.

Additionally, Spearman correlation analysis indicated that in the NBTL-H group, the abundance of *g_Prevotellaceae_Ga6A1_group* (Figure 6D, *r* = −0.82, *p* = 0.0066) and *s_uncultured_bacterium_g_Prevotellaceae_Ga6A1_group* (Figure 6G, *r* = −0.82, *p* = 0.0066) were negatively correlated with miR-20a-5p, suggesting that the reduction of these bacterial groups may promote the expression of miR-20a-5p. Conversely, the abundance of *s_Metamycoplasma_sualvi* (Figure 6E, *r* = 0.83, *p* = 0.0053) showed a positive correlation with miR-20a-5p, indicating that the increase of this beneficial bacterium might enhance the production of miR-20a-5p. Although the abundance of *f_Mycoplasmataceae* (Figure 6C, *r* = 0.27, *p* = 0.48) and *s_uncultured_bacterium_g_norank_f_Eggerthellaceae* (Figure 6F, *r* = 0.44, *p* = 0.24) was positively correlated with miR-20a-5p, the correlations were not significant, but they still hold potential biological relevance.

Similarly, in the NBTL-H group, *g_Prevotellaceae_Ga6A1_group* (Figure 6K, *r* = −0.78, *p* = 0.013) and *s_uncultured_bacterium_g_Prevotellaceae_Ga6A1_group* (Figure 6N, *r* = −0.76, *p* = 0.017) both exhibited a strong negative correlation with miR-223-3p. Conversely, *s_Metamycoplasma_sualvi* (Figure 6L, *r* = 0.71, *p* = 0.032) was positively correlated with miR-223-3p. Although the correlations of *f_Mycoplasmataceae* (Figure 6J, *r* = 0.45, *p* = 0.22) and *s_uncultured_bacterium_g_norank_f_Eggerthellaceae* (Figure 6M, *r* = 0.66, *p* = 0.053) with miR-223-3p were not significant, they still possess potential biological importance.

### 3.7 Deep learning-driven prediction and rat model validation of NBTL’s inhibition of the VEGF pathway via characteristic gut microbiota features

**Figure 7:**
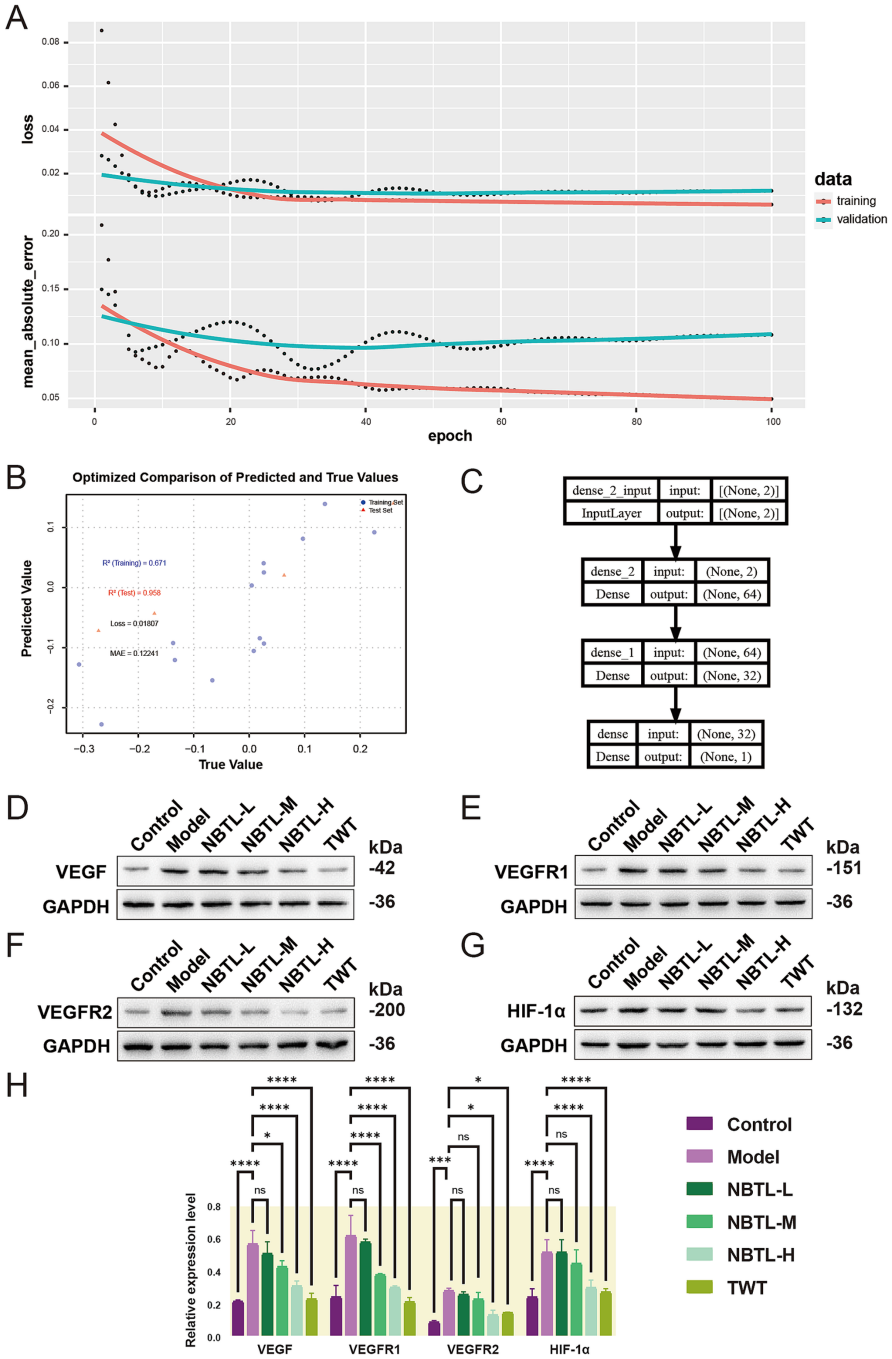
Deep learning-driven prediction and rat model validation of NBTL’s inhibition of the VEGF pathway via characteristic gut microbiota features. **(A)** The figure illustrates the changes in training and validation loss as well as mean absolute error over epochs. The top panel shows the loss curves, with red and blue lines representing the training and validation datasets, respectively. Dots mark specific values at each epoch, while the curves indicate the smoothed trends. The bottom panel depicts the mean absolute error, with red and blue lines corresponding to the training and validation datasets. **(B)** The scatter plot compares predicted vs. actual values, with blue dots for training data and red triangles for validation data. The *R*^2^ values are 0.671 (training) and 0.958 (validation), with a loss of 0.01807 and a MAE of 0.12241. **(C)** The network architecture diagram displays the model’s structure, from input to output layers, with two input features processed through dense layers before outputting a single prediction. **(D–G)** Show the expression levels of VEGF, VEGFR1, VEGFR2, and HIF-1α proteins in rats. **(H)** Shows the relative levels of these proteins. The groups include control (healthy control), model (untreated disease model), NBTL-L/M/H (low, medium, and high doses of new bitongling), and TWT (Tripterygium wilfordii positive control), with GAPDH used as a loading control. **(A–C)** Sample size per group: *n* = 6. **(D–H)** Sample size per group: *n* = 3. ^*^p < 0.05, ^***^p < 0.001, and ^****^p < 0.0001, and ns for non-significant differences.

We initially employed a deep neural network (DNN) to predict how NBTL inhibits VEGF-driven angiogenesis by analyzing five characteristic gut microbiota features. The training process (Figure 7A) showed a rapid decline and subsequent stabilization in the model’s loss and error rates, with validation data performing slightly worse than the training data, indicating good generalization. Further optimization (Figure 7B) revealed an *R*^2^ of 0.671 for the training set and a high *R*^2^ of 0.958 for the validation set, with a loss of 0.01807 and a MAE of 0.12241, underscoring the model’s robust predictive capability regarding NBTL’s regulation of the VEGF pathway. The model architecture, from input to output layers, effectively captured complex relationships within the data through multiple layers of feature extraction (Figure 7C).

Building on the DNN model’s predictions, we further validated NBTL’s inhibitory effects on VEGF signaling using a rat model. Western blot analysis of VEGF, VEGFR1, VEGFR2, and HIF-1α protein expression demonstrated a dose-dependent inhibitory effect of NBTL across low, medium, and high dosage groups (Figures 7D–G). The high-dose group showed a highly significant suppression of VEGF, VEGFR1, VEGFR2, and HIF-1α expression (*p* < 0.05 and *p* < 0.0001), with the medium dose also significantly reducing VEGF, VEGFR1, and HIF-1α levels (*p* < 0.05 and *p* < 0.0001) (Figure 7H). In conclusion, NBTL not only exhibited strong potential in inhibiting the VEGF signaling pathway in predictive models but also demonstrated substantial efficacy in rat models, particularly at higher doses, supporting its potential use in treating rheumatoid arthritis.

### 3.8 NBTL medicated serum suppresses HUVEC viability, migration, and angiogenesis via miRNA modulation and VEGF signaling pathway inhibition: validation at the cellular level with deep learning predictive models

**Figure 8:**
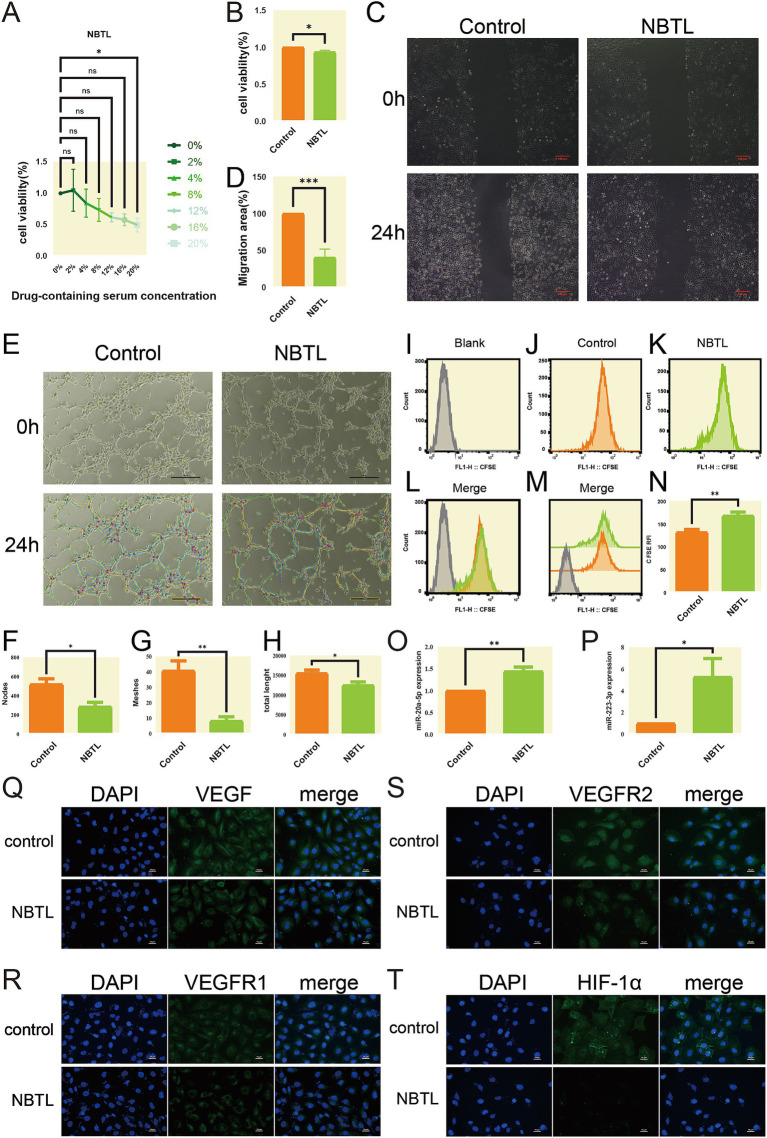
NBTL medicated serum suppresses HUVEC viability, migration, and angiogenesis via miRNA modulation and VEGF signaling pathway inhibition: validation at the cellular level with deep learning predictive models. **(A)** Dose-response curve of cell viability treated with NBTL-medicated serum. The *x*-axis represents different concentrations of NBTL-medicated serum (0, 2, 4, 8, 12, 16, 20%), and the *y*-axis represents cell viability percentage. **(B)** Comparison of cell viability between the control group (orange, control) and NBTL-medicated serum group (green, NBTL). ^*^p < 0.05, ns: not significant. **(C)** Wound healing assay showing the effect of NBTL-medicated serum on HUVEC cell migration at 0 and 24 h. Red scale bars = 100 μm. **(D)** Quantification of HUVEC cell migration area after 24 h; ^***^p < 0.001. **(E)** Tube formation assay images of HUVEC cells. Control group (left) and NBTL group (right) with unprocessed (top) and software-analyzed images (bottom). Black scale bars = 100 μm. **(F–H)** Quantitative analysis of tube formation: number of nodes, meshes, and total tube length. ^*^p < 0.05 and ^**^p < 0.01. **(I–N)** CFSE staining to assess cell proliferation: **(I)** unlabeled HUVEC cells (blank) for baseline; **(J)** control group; **(K)** NBTL group; **(L–M)** overlay of CFSE intensity between control and NBTL groups; **(N)** statistical analysis of CFSE fluorescence intensity. ^**^p < 0.01. **(O,P)** Expression levels of miR-20a-5p **(O)** and miR-223-3p **(P)** in HUVEC cells, control vs. NBTL. ^*^p < 0.05 and ^**^p < 0.01. **(Q–T)** Immunofluorescence staining of HUVEC cells for VEGF **(Q)**, VEGFR1 **(R)**, VEGFR2 **(S)**, and HIF-1α **(T)**, with DAPI (blue) marking nuclei and specific markers (green) showing protein expression. White scale bars = 10 μm. Control = blank serum, NBTL = NBTL-medicated serum.

In this study, we utilized the CCK-8 assay to evaluate the effects of NBTL medicated serum on the viability of HUVECs. The results indicated a dose-dependent decrease in cell viability as the concentration of NBTL medicated serum increased from 0 to 20%, demonstrating an inhibitory effect on cell growth (Figure 8A). At the highest concentration (20%), cell viability significantly decreased relative to the 0% group (*p* < 0.05). A comparison between the control group (20% blank serum) and the NBTL medicated serum group (20%) further confirmed that NBTL significantly reduced cell viability (*p* < 0.05) (Figure 8B), establishing 20% as the optimal inhibitory concentration for subsequent experiments.

Next, we assessed the impact of NBTL medicated serum on HUVEC migration using a scratch assay. After 24 h of incubation, the control group displayed significant wound closure, while the NBTL-treated group showed limited closure, indicating a substantial inhibition of cell migration (Figures 8C,D, *p* < 0.001). This suggests that NBTL may exert its anti-angiogenic effects, in part, by inhibiting cell migration.

We also conducted a tube formation assay to investigate the effect of NBTL medicated serum on angiogenesis. Microscopic observations revealed that HUVECs in the control formed more tube-like structures than in the NBTL group, where these structures were notably fewer (Figures 8E–H, *p* < 0.05 and *p* < 0.01), confirming its anti-angiogenic properties.

Additionally, we analyzed the effect of NBTL medicated serum on HUVEC proliferation using CFSE staining and flow cytometry. The results demonstrated a marked decrease in cell proliferation in the NBTL group relative to the control (Figures 8I–N, *p* < 0.01), supporting its role in inhibiting cell growth.

Quantitative PCR (qPCR) analysis assessed the expression of miR-20a-5p and miR-223-3p, two miRNAs linked to angiogenesis inhibition. The data showed that NBTL-treated serum significantly upregulated these miRNAs (Figures 8O,P, *p* < 0.05 and *p* < 0.01), indicating a possible mechanism through which NBTL inhibits angiogenesis.

Finally, immunofluorescence staining demonstrated that NBTL medicated serum significantly downregulated the expression of VEGF, VEGFR1, VEGFR2, and HIF-1α proteins, which are key components of the VEGF signaling pathway in HUVECs (Figures 8Q–T).

In conclusion, this study systematically demonstrates that NBTL medicated serum significantly inhibits HUVEC proliferation, migration, and angiogenesis through multiple mechanisms, offering a potential therapeutic application in anti-angiogenesis treatment.

## 4 Discussion

**Figure 9:**
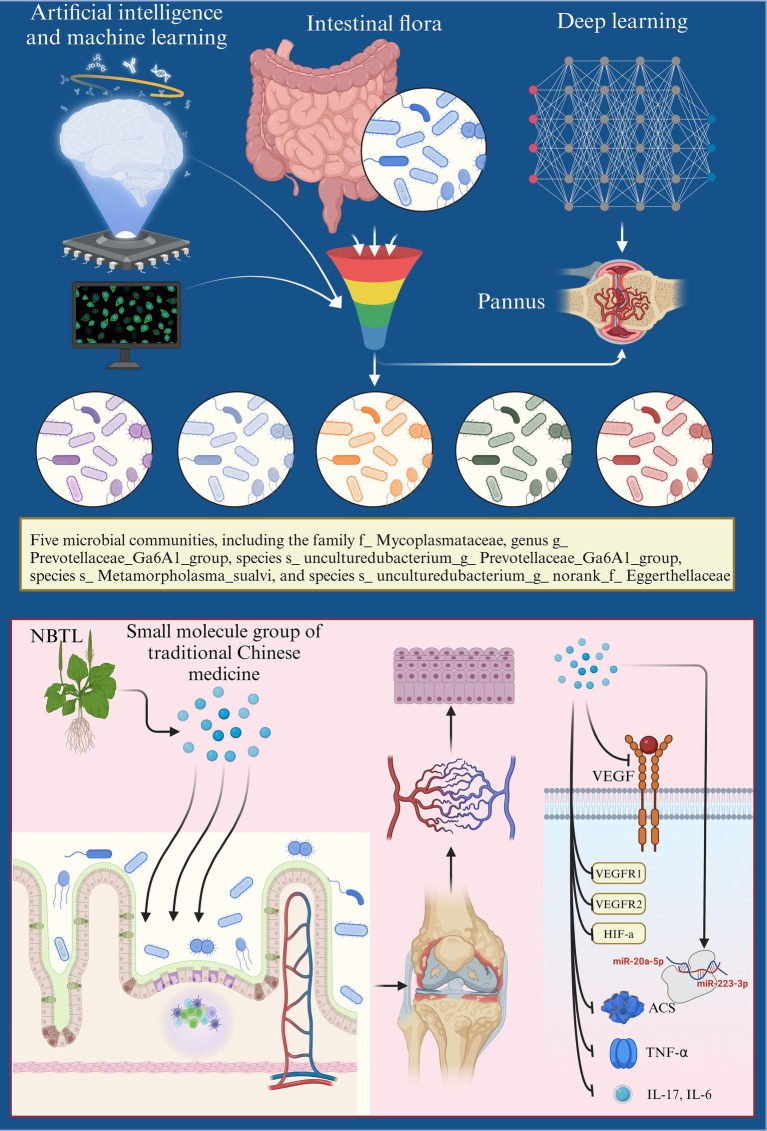
Mechanism diagram: integrating machine learning and deep neural networks to analyze the role of NBTL in modulating angiogenesis in rheumatoid arthritis via the gut-joint axis.

Our evaluation centered on uncovering the underlying mechanisms, particularly gut microbiota modulation and VEGF angiogenesis pathway inhibition. The findings demonstrate that NBTL not only significantly alleviates RA symptoms by suppressing key inflammatory factors but also profoundly modulates gut microbiota composition, highlighting the critical roles of specific bacterial taxa like *f_Mycoplasmataceae* and *g_Prevotellaceae_Ga6A1_group* in RA treatment. Furthermore, NBTL inhibits the VEGF angiogenesis signaling pathway by promoting the expression of anti-angiogenic factors, including miR-20a-5p and miR-223-3p, while simultaneously downregulating the expression of VEGF, VEGFR1, VEGFR2, and HIF-1α, thereby reducing angiogenesis in the synovial tissue. The Mechanism diagram (Figure 9) visually encapsulates how NBTL inhibits angiogenesis through the gut-joint axis, paving the way for future advancements in precision medicine and personalized therapeutic strategies.

Firstly, NBTL showed notable anti-inflammatory effects in the RA model. NBTL treatment markedly lowered TNF-α, IL-17, and IL-6 levels compared to the control, particularly in the medium- and high-dose groups, demonstrating a clear dose-dependence. These cytokines are key drivers in RA pathogenesis, with their overexpression strongly associated with joint inflammation and damage to cartilage and bone (Yamanaka, 2015; Taams, 2020; Kishimoto and Kang, 2022; Hashizume et al., 2008). By inhibiting these key cytokines, NBTL effectively attenuated the inflammatory response, suggesting a primary mechanism through which it may exert its therapeutic effects in RA. Additionally, the study highlighted the potential involvement of ASC in RA pathogenesis. ASC levels were notably elevated in the synovial tissue of RA rats, but high-dose NBTL treatment significantly reduced its expression. This finding not only underscores the complex role of ASC in RA progression but also opens new avenues for understanding NBTL’s mechanisms, indicating that it may modulate inflammation through an inflammasome-independent pathway (Ippagunta et al., 2010).

Secondly, gut microbiota is crucial in RA pathogenesis. Alterations in gut microbial communities, including the emergence of rare taxa and reduced microbial diversity, have been implicated in the development of RA (Zaiss et al., 2021). Our study demonstrated that NBTL significantly alters gut microbiota in RA rats, boosting beneficial bacteria like *f_Mycoplasmataceae*, *s_Metamycoplasma_sualvi*, and *s_uncultured_bacterium_g_norank_f_Eggerthellaceae*, while decreasing potential pathogens such as *g_Prevotellaceae_Ga6A1_group* and *s_uncultured_bacterium_g_Prevotellaceae_Ga6A1_group*. The reduction of *g_Prevotellaceae_Ga6A1_group* is particularly notable, as it suggests a possible role in RA pathology, with NBTL potentially exerting therapeutic effects by modulating these specific bacterial taxa. This discovery not only sheds light on the mechanisms of NBTL but also opens new avenues for exploring the RA-gut microbiota relationship in future research.

Our study also applied advanced machine learning (ML) techniques, such as SVM-RFE and random forest, to evaluate NBTL’s impact on gut microbiota in the RA rat model. SVM-RFE effectively identified the most informative features (Sanz et al., 2018), while random forest demonstrated strong nonlinear modeling capabilities in processing complex data (Zhang et al., 2021; Roguet et al., 2018; Belk et al., 2018). However, each method has its strengths and limitations. SVM-RFE may lose some information when dealing with complex biological data, and the inherent randomness in random forest could lead to instability in feature selection. Despite these limitations, the overlap and divergence in microbial selection between the two methods suggest that these microbes may be crucial to NBTL’s therapeutic effects. We recommend the combined use of these techniques in future research, with particular attention to microbes identified by only one method, as they may harbor undiscovered biological significance. To ensure the robustness and generalizability of the models, we employed 5-fold cross-validation with five repetitions to evaluate both the SVM-RFE and random forest models. This rigorous cross-validation approach minimized the risk of overfitting and confirmed the stability of the results across different data subsets. Additionally, while these models performed well on the current dataset, their generalizability to larger and more diverse datasets remains a critical area for future research. Expanding the dataset to include samples with varying microbiota compositions could help further validate the predictive accuracy of the identified microbial signatures and ensure the models’ broader applicability across different populations. Furthermore, it is important to acknowledge that both SVM-RFE and random forest have their own limitations in handling complex biological data. For instance, SVM-RFE may suffer from information loss when dealing with high-dimensional, nonlinear feature relationships, while random forest’s inherent randomness can cause variability in feature selection. However, these issues were mitigated by combining both methods, allowing for cross-validation of the microbial taxa identified and reinforcing the reliability of our findings. Future studies should consider using additional machine learning techniques, such as ensemble models or deep learning algorithms, to further enhance model stability and prediction accuracy. In light of these considerations, we recommend that future research incorporate larger and more diverse datasets to assess the robustness of these models in varied clinical or experimental contexts. Moreover, we suggest exploring other machine learning approaches, such as neural networks or support vector machines, to address the potential weaknesses of the current methods and identify novel microbial markers that may have been overlooked by a single method.

Through Spearman correlation analysis, we further identified significant associations between NBTL-regulated key microbial taxa and angiogenesis, leading to the hypothesis that these taxa could predict NBTL’s efficacy in inhibiting the VEGF angiogenesis pathway. Based on this hypothesis, we developed and optimized a deep neural network (DNN) model that effectively predicted NBTL’s potential anti-angiogenic mechanisms through modulation of specific gut microbiota. This model demonstrated high accuracy in predicting unknown data, providing a powerful analytical tool and theoretical foundation for understanding NBTL’s mechanisms.

It is worth noting that while NBTL significantly inhibited the VEGF signaling pathway, its dual inhibition of VEGFR1 and VEGFR2 deviates from the conventional understanding of these receptors’ opposing expression trends. Traditionally, VEGFR2 is considered the primary pro-angiogenic factor, while VEGFR1 has a regulatory role, with their expressions typically showing inverse trends (Pérez-Gutiérrez and Ferrara, 2023; Simons et al., 2016; Chiodelli et al., 2017). The ability of NBTL to concurrently inhibit both receptors suggests a non-typical mechanism of action, offering a new perspective on its therapeutic efficacy and indicating a more comprehensive regulation of the VEGF pathway by NBTL.

In addition, our study further highlights the therapeutic relevance of miR-20a-5p and miR-223-3p in RA. Recent research indicates that miR-223-3p is commonly dysregulated in RA patients and is involved in modulating inflammatory, with its expression correlating with disease activity and synovial inflammation (Wu et al., 2020; Dunaeva et al., 2018; Moriya et al., 2017). Although miR-20a-5p has not been extensively studied in RA, its established role in VEGF regulation suggests potential therapeutic value (Guo et al., 2021). These findings open avenues for further exploration of these miRNAs as biomarkers or therapeutic targets for RA. However, additional clinical studies involving patient samples are needed to validate their roles and clarify their potential applications.

To further substantiate the molecular mechanisms underpinning NBTL’s therapeutic effects, we employed UPHLC-MS-TOF to analyze the prototype components and metabolites in the blood. This analysis revealed that the bioactivities observed, including anti-inflammatory and anti-angiogenic effects, are closely linked to specific active ingredients and their metabolites. These findings reinforce the proposed mechanisms of action, suggesting that the pharmacokinetics of NBTL’s constituents contribute significantly to its overall efficacy. This aspect of the study provides additional evidence for NBTL’s role in modulating biological pathways relevant to rheumatoid arthritis treatment.

Additionally, we employed PLS-DA instead of the traditional principal component analysis (PCA) for analyzing both gut microbiome and transcriptome data. Compared to PCA, PLS-DA demonstrated clear advantages in distinguishing between different experimental conditions, such as treatment and control groups, as it considers not only the main directions of data variance but also their relationships with the response variables (Ruiz-Perez et al., 2020; Lasalvia et al., 2022; Lee et al., 2018). However, when sample size is limited or there are many variables, PLS-DA may increase model complexity, potentially leading to overfitting and reduced generalizability. Additionally, PLS-DA results may be less intuitive to interpret compared to PCA. Therefore, careful parameter selection is crucial when applying PLS-DA models. To optimize this process, we plan to further evaluate and refine the parameter selection and validation procedures for PLS-DA in future studies, aiming to ensure model stability while maximizing the extraction and utilization of biological information from both gut microbiome and transcriptome data.

The doses of NBTL used in this study were carefully selected based on preclinical studies and equivalent human dosage extrapolation. The low, medium, and high doses were chosen to evaluate a dose-response relationship and assess the potential therapeutic window. Among these, the medium dose of NBTL demonstrated optimal efficacy in alleviating RA symptoms, while avoiding significant adverse effects. This dosage range, extrapolated from preclinical data, provides a reasonable starting point for future clinical trials. However, further studies are needed to determine the precise dose that balances efficacy and safety in humans. These findings could serve as a foundation for subsequent clinical investigations to establish an optimal dosing regimen for NBTL in rheumatoid arthritis treatment.

While NBTL shows promising therapeutic potential, clinical application faces challenges, particularly regarding safety and regulatory hurdles. Long-term safety needs to be evaluated, as prolonged use of herbal formulations can sometimes cause toxicity. Further preclinical studies on chronic toxicity are necessary. Additionally, due to NBTL’s multi-component nature, regulatory agencies will require detailed documentation on its efficacy, safety, and consistent manufacturing. Establishing clear relationships between dose and effect, along with standardized quality control, will be crucial for regulatory approval. In summary, NBTL holds significant potential for RA treatment, but addressing safety concerns, regulatory requirements, and standardization will be key for its clinical translation. Future studies should focus on determining optimal dosing, ensuring long-term safety, and identifying biomarkers for personalized treatment.

Despite the extensive documentation of the association between gut microbiota dysbiosis and rheumatoid arthritis (RA), our study offers novel insights by introducing new bitongling (NBTL) as a unique traditional Chinese medicine (TCM) formulation with a distinct herbal composition. Unlike previously studied TCM formulas such as Gegen Qinlian Decoction (Hu et al., 2024; Lv et al., 2019; Peng et al., 2024), NBTL comprises a specific combination of herbs including *Cinnamomi ramulus*, *Saposhnikoviae radix*, *Ephedrae herba*, *Sinomenii caulis*, *Aconiti radix*, and *Vespae Nidus*. This unique formulation has not been extensively explored in the context of RA and gut microbiota modulation, thereby providing a fresh perspective on TCM-based therapeutic strategies.

Furthermore, our integrative approach combining gut microbiota 16S rDNA sequencing, transcriptomic analysis, and advanced machine learning techniques (SVM-RFE, random forest, and deep neural networks) allows for a comprehensive exploration of the regulatory mechanisms linking gut microbiota modulation and the VEGF angiogenesis pathway. This multi-omics and computational methodology surpasses previous studies by providing a more holistic understanding of how NBTL influences RA pathogenesis. Specifically, the identification and validation of specific microbial taxa (e.g., *f_Mycoplasmataceae*, *g_Prevotellaceae_Ga6A1_group*) and miRNAs (miR-20a-5p and miR-223-3p) associated with NBTL treatment efficacy offer potential biomarkers for personalized medicine approaches in RA treatment.

Additionally, our study reveals that NBTL concurrently inhibits both VEGFR1 and VEGFR2, a finding that deviates from the conventional focus on VEGFR2 as the primary pro-angiogenic factor in RA. This dual inhibition suggests a more comprehensive regulatory effect on the VEGF signaling pathway, potentially enhancing the anti-angiogenic and anti-inflammatory efficacy of NBTL compared to therapies targeting individual components of this pathway.

Moreover, the use of machine learning models to predict and validate the regulatory relationships between gut microbiota and the VEGF pathway represents an innovative aspect of our research. These computational tools facilitate the identification of key microbial and molecular players in RA, thereby enhancing the translational potential of our findings and paving the way for future precision medicine and personalized therapeutic strategies.

## Data Availability

The 16S rRNA sequencing data generated for this study have been deposited in the Zenodo repository. The dataset is publicly accessible at https://zenodo.org/records/14679790.

## Footnotes

^1^ https://string-db.org/

## Glossary

RA: Rheumatoid arthritis
DMARDs: Disease-modifying antirheumatic drugs
TCM: Traditional Chinese medicine
NBTL: New bitongling
VEGF: Vascular endothelial growth factor
CIA: Collagen-induced arthritis
16S rDNA: 16S ribosomal DNA
GSVA: Gene set variation analysis
TNF-α: Tumor necrosis factor alpha
IL: Interleukin
ASC: Apoptosis-associated speck-like protein containing a CARD
UPHLC-MS-TOF: Ultra-performance high-performance liquid chromatography coupled with time-of-flight mass spectrometry
HE: Hematoxylin and eosin
ELISA: Enzyme-linked immunosorbent assay
SDS-PAGE: Sodium dodecyl sulfate-polyacrylamide gel electrophoresis
PVDF: Polyvinylidene difluoride
GAPDH: Glyceraldehyde 3-phosphate dehydrogenase
HIF-1α: Hypoxia-inducible factor 1-alpha
qPCR: Quantitative polymerase chain reaction
OTU: Operational taxonomic units
LEfSe: Linear discriminant analysis effect size
SVM-RFE: Support vector machine-recursive feature elimination
AUC: Area under the curve
ROC: Receiver operating characteristic
WGCNA: Weighted gene co-expression network analysis
PPI: Protein–protein interaction
DNN: Deep neural network
HUVECs: Human umbilical vein endothelial cells
RPMI: Roswell Park Memorial Institute
FBS: Fetal bovine serum
CCK-8: Cell Counting Kit-8
CFSE: Carboxyfluorescein diacetate succinimidyl ester
PCA: Principal component analysis
PLS-DA: Partial least squares discriminant analysis
MAE: Mean absolute error
