# Supplementary material for: New bitongling regulates gut microbiota to predict angiogenesis in rheumatoid arthritis via the gut-joint axis: a deep neural network approach
Source: Front Microbiol. 2025 Feb 3;16:1528865. doi: 10.3389/fmicb.2025.1528865 (PMC11830818; doi:10.3389/fmicb.2025.1528865)
Supplement: Supplementary file 1 [file Supplementary_file_1.docx]

**Supplement 1. Data information summary**

| **Data type** | **Sample** | | **Statistical Power** |
| --- | --- | --- | --- |
| **Gut Microbiota** | **Control** | **8** | **0.9233** |
|  | **Model** | **8** |  |
|  | **NBTL-H** | **8** |  |
| **Transcriptome** | **Control** | **6** | **0.8193** |
|  | **Model** | **6** |  |
|  | **NBTL-H** | **6** |  |
