# Supplementary material for: New bitongling regulates gut microbiota to predict angiogenesis in rheumatoid arthritis via the gut-joint axis: a deep neural network approach
Source: Front Microbiol. 2025 Feb 3;16:1528865. doi: 10.3389/fmicb.2025.1528865 (PMC11830818; doi:10.3389/fmicb.2025.1528865)
Supplement: Supplementary file 2 [file Supplementary_file_2.docx]

|  | **Performance Summary** | |
| --- | --- | --- |
| **Metric** | **Dataset** | **Value** |
| **RMSE** | **Training_Set** | **0.084** |
| **Shapiro-Wilk p-value (Residual Normality)** | **Training_Set** | **0.124** |
| **Pearson Correlation (R)** | **Training_Set** | **0.819** |
| **RMSE** | **Test_Set** | **0.134** |
| **Shapiro-Wilk p-value (Residual Normality)** | **Test_Set** | **0.640** |
| **Pearson Correlation (R)** | **Test_Set** | **0.979** |

**Supplement 2. Statistical Performance Metrics: RMSE, Residual Normality Test, and Correlation**
